# A Possible Trifunctional β-Carotene Synthase Gene Identified in the Draft Genome of *Aurantiochytrium* sp. Strain KH105

**DOI:** 10.3390/genes9040200

**Published:** 2018-04-09

**Authors:** Hiroaki Iwasaka, Ryo Koyanagi, Ryota Satoh, Akiko Nagano, Kenshi Watanabe, Kanako Hisata, Noriyuki Satoh, Tsunehiro Aki

**Affiliations:** 1 Department of Molecular Biotechnology, Graduate School of Advanced Sciences of Matter, Hiroshima University, Higashi-Hiroshima 739-8530, Japan; wdzra2nhph@gmail.com (H.I.); ctsak9y9g9@gmail.com (R.S.); pnsptrui35@gmail.com (A.N.); kwatanabe@hiroshima-u.ac.jp (K.W.); 2 Marine Genomic Unit, Okinawa Institute of Science and Technology Graduate University, Onna, Okinawa 904-0945, Japan; koyanagi@oist.jp (R.K.); kanako@oist.jp (K.H.); 3 JST-CREST, Saitama 332-0012, Japan

**Keywords:** *Aurantiochytrium* sp. strain KH105, genome sequencing, carotenoid biosynthesis, trifunctional enzyme gene, *CrtIBY*

## Abstract

Labyrinthulomycetes have been regarded as a promising industrial source of xanthophylls, including astaxanthin and canthaxanthin, polyunsaturated fatty acids such as docosahexaenoic acid and docosapentaenoic acid, ω-3 oils, and terpenic hydrocarbons, such as sterols and squalene. A Thraustochytrid, *Aurantiochytrium* sp. KH105 produces carotenoids, including astaxanthin, with strong antioxidant activity. To gain genomic insights into this capacity, we decoded its 97-Mbp genome and characterized genes for enzymes involved in carotenoid biosynthesis. Interestingly, all carotenogenic genes, as well as other eukaryotic genes, appeared duplicated, suggesting that this strain is diploid. In addition, among the five genes involved in the pathway from geranylgeranyl pyrophosphate to astaxanthin, geranylgeranyl phytoene synthase (*crtB*), phytoene desaturase (*crtI*) and lycopene cyclase (*crtY*) were fused into single gene (*crtIBY*) with no internal stop codons. Functionality of the trifunctional enzyme, CrtIBY, to catalyze the reaction from geranylgeranyl diphosphate to β-carotene was confirmed using a yeast assay system and mass spectrometry. Furthermore, analyses of differential gene expression showed characteristic up-regulation of carotenoid biosynthetic genes during stationary and starvation phases under these culture conditions. This suggests genetic engineering events to promote more efficient production of carotenoids. We also showed an occurrence of *crtIBY* in other Thraustochytrid species.

## 1. Introduction

A marine microbe, *Aurantiochytrium*, belongs to the order Thraustochytriaceae (so-called Thraustochytrids), pertaining to the class Labyrinthulomycetes, of the phylum Heterokonta, within the kingdom Chromista [1,2]. Thraustochytrids are a group of non-photosynthetic, marine, fungoid protists, characterized by an ectoplasmic net and a cell wall composed of non-cellulosic, sulfated scales [1,2,3]. It has been argued that the biomass of Labyrinthulomycetes in the water column may equal or even exceed that of bacteria [4]. Their high biomass and production of degradative enzymes indicate that Labyrinthulomycetes play a significant ecological role as alternative food sources for picoplankton feeders, as well as being active decomposers and consumers in marine microbial food chains [5].

Labyrinthulomycetes have also been regarded as a promising industrial source of xanthophylls, including astaxanthin and canthaxanthin, polyunsaturated fatty acids (PUFA) such as docosahexaenoic acid (DHA; 22:6n-3) and docosapentaenoic acid (DPA; 22:5n-6), ω-3 oils [6], and terpenic hydrocarbons, such as sterols and squalene [7,8,9]. In a previous study, we isolated and characterized *Aurantiochytrium* sp. strain KH105 as a potential source of carotenoids [10]. This strain produces β-carotene as well as xanthophylls such as echinenone, canthaxanthin, phoenicoxanthin, and astaxanthin [10,11]. The intracellular composition of these carotenoids is affected by media and culture conditions. However, the biosynthetic mechanism of these carotenoids has not been elaborated. Recently, genomes of several Thraustochytrid species have been decoded [12,13,14,15]. Here, we decoded the genome of *Aurantiochytrium* sp. strain KH105 specifically to understand its capacity to synthesize carotenoids.

## 2. Materials and Methods

### 2.1. Microorganism

*Aurantiochytrium* sp. strain KH105 [11], formerly classified in the genus *Schizochytrium*, but amended later [3], was maintained on GPY agar medium composed of 3% glucose, 0.6% hipolypeptone (Nihon Pharmaceutical, Tokyo, Japan), 0.2% yeast extract (Kyokuto Pharmaceutical Industrial, Tokyo, Japan), and 2% sea salts (Merck KGaA, Darmstadt, Germany), pH 7.0, with 1.8% agar at 28 °C, and pre-cultured in GPY liquid medium at 300 rpm for up to 24 h. A baffled 200-mL flask with 50 mL of GPY medium was inoculated with 1 mL of pre-cultured medium and cultured at 28 °C, 160 rpm. DNA extraction was performed on cells incubated for 96 h at 28 °C, and RNA extraction from cells cultured at 28 °C for 27 h (mid-log phase), 40 h (late-log to stationary phase) and 48 h (starvation phase), respectively.

### 2.2. DNA Preparation

Cells were harvested from culture broth by centrifugation (10,000× *g*, 20 min at 4 °C), and frozen in liquid nitrogen. Nuclear DNA for sequencing was obtained using a protease, RNAase, and a phenol-chloroform extraction protocol [16]. Packed, frozen cells were pulverized using a sterile mortar and pestle in liquid nitrogen. The powder was treated with 2% cetyltrimethylammonium bromide (CTAB) solution (2% CTAB, 100 mM Tris-HCl, pH 8.0, 20 mM ethylenediaminetetraacetic acid (EDTA), 1.4 M NaCl) for 1 h at 65 °C. Solutions were centrifuged at 1500× *g* for 5 min at 4 °C. Supernatant was subjected to 20 μg/mL RNase treatment (37 °C, 30 min) and further treated by adding an equal volume of chloroform/isoamyl alcohol (24:1, *v*/*v*) with gentle rotation for 1 h. After centrifugation at 1500× *g* using Phase lock gel tubes (Funakoshi, Tokyo, Japan), supernatants were transferred to new tubes. An additional chloroform treatment was repeated twice. By adding 1.5× volumes of 1% CTAB, genomic DNA was precipitated. Precipitated DNA was treated with 0.1 mg/mL proteinase K (Takara Bio Inc., Shiga, Japan) in 1 M NaCl for 2–3 h at 56 °C. By adding at least 2.5× volumes of ethanol, DNA was precipitated again. After rinsing with 70% ethanol twice, DNA was dissolved overnight in 10 mM Tris-HCl, 1 mM EDTA, pH 7.4, at 4 °C.

### 2.3. Genome Sequencing and Assembly

Genomic DNA was fragmented, and libraries were prepared and sequenced according to the manufacturer’s protocols with whole-genome shotgun (WGS) reads using a Roche 454 GS-FLX (Roche Diagnostics, Basel, Switzerland) [17], and Illumina Miseq and GAIIx sequencers (Illumina, San Diego, CA, USA) [18]. To obtain contigs, 454 and Miseq paired-end reads (Illumina, San Diego, CA, USA) were combined and assembled de novo using GS De Novo Assembler version 2.6 (Newbler, Roche, Basel, Switzerland) [17]. Then, subsequent scaffolding was performed with SOPRA [19] and SSPACE [20] using Illumina mate-pair information. At least five consistent read-pairs were needed to form a connection. Gaps inside scaffolds were closed with Illumina paired-end data using GapCloser [21]. Genome size can be roughly estimated by the k-mer value of new-generation sequence reads. Illumina sequences were quality trimmed (QV ≥ 20) and possible polymerase chain reaction (PCR) duplicates were removed with MarkDuplicates in Picard tools (http://picard.sourceforge.net, Broad Institute, Cambridge, MA, USA). K-mers (length: 21, 31, 41, 51, 61, and 71) in the dataset were counted using JELLYFISH [22].

### 2.4. RNA Sequencing Analyses

We performed transcriptome sequencing using an Illumina GAIIx sequencer (Illumina, San Diego, CA, USA). RNA was isolated from cells at three time-points (27, 40, and 48 h) during culturing at 28 °C, as described above. At each time-point, three samples were taken from different flasks. After washing cells with distilled water, total RNA was extracted following the manufacturer’s instructions (Invitrogen, Carlsbad, CA, USA) and purified using DNase and RNeasy micro kits (QIAGEN, Hilden, Germany). Transcriptome libraries for Illumina GAIIx were prepared with TruSeq Standard mRNA LT Sample Prep kit (Illumina, San Diego, CA, USA) and sequenced as per manufacturer’s instruction (paired-end sequences, 150 bp ×2). A total of 113.6 Gbp nucleotide (Gbn) sequence data were obtained, 71.8% of which (81.6 Gbn) had a quality value ≥30. These high-quality sequences were assembled with the Velvet/Oases assembler with a hash length of 37 [23]. Trinity assemblies were also used [24]. As a result, we obtained 112,852 contigs (29.3 Mbp) over 100 bp with an N50 size of 1575 bp. Of these, 88,570 (78%) had a blat alignment to the assembled genome (with default settings). A total of 68,571 of the 88,570 contigs were found to have complete open reading frames (ORFs) from the start to stop codon of at least 450 bp. Of these putatively full-length RNA contigs, 66,342 (97.3%) had a blat alignment to the assembled scaffolds. These data were used to produce gene models and annotation.

### 2.5. Gene Modeling

Gene predictions (*Aurantiochytrium* KH105 Gene Model ver. 1) were performed using ab initio and complementary DNA (cDNA) sequence alignment prediction derived from Augustus software [25]. Assembled *Aurantiochytrium* transcriptome sequences were processed with PASA [26], and the 500 longest ORFs were used to train the prediction software. Assembled Illumina RNA sequencing (RNA-seq) data were aligned to the assembled genome and incorporated as an Augustus “hint” on a repeat-masked genome produced using RepeatMasker [27].

### 2.6. Genome Browser

A genome browser was established for the assembled sequences using the Generic Genome Browser (GBrowser) 2.17 [28]. 

### 2.7. Annotation and Identification of Aurantiochytrium Genes

Protein-coding genes in the *Aurantiochytrium* KH105 genome were surveyed as follows. (i) Nucleotide and amino acid sequences of well annotated genes of model organisms were used as queries for basic local alignment search tool (BLAST) searches including TBLASTN (expect value < 1 × 10^−10^) of the *Aurantiochytrium* KH105 genome; (ii) Pfam domain searches were performed to identify protein domains included in putative proteins from all gene models (Pfam-A.hmm, release 24.0; http://pfam.sanger.ac.uk) [29]. *Aurantiochytrium* genes with conserved domains were included in a phylogenetic analysis; (iii) In order not to miss divergent homologs specific to Labyrinthula lineages, we confirmed them with at least one gene hit using reciprocal BLAST searches. Divergent homologs were shown as outgroups of annotated genes. RNA-seq reads were mapped to the assembled genome [29]. MAKER2 was used to tune-up the annotation and modeling [30].

### 2.8. Molecular Phylogeny

Molecular phylogenic analyses of phytoene desaturase (CrtI), geranylgeranyl phytoene synthase (CrtB), and geranylgeranyl phytoene synthase (CrtY) domains of CrtIBY were carried out using ClustalW [31]. Their amino acid sequences were aligned using default parameters and the resulting datasets were used to visualize maximum-parsimony trees with Sea View [32].

### 2.9. Analyses of Gene Expression Profiles

Differential gene expression profiles were analyzed using RNA-seq data with TopHat and Cluffinks, according to the method of Trapnell et al. [33]. The expression level of each gene was calculated as fragments per kilobase of transcript per million mapped reads (FPKM) with Cufflinks [34].

### 2.10. Functional Assay of crtIBY

After cultivation of *Aurantiochytrium* sp. KH105 in GPY medium for 4 days, total genomic DNA was extracted from cells using the phenol-chloroform method. The *crtIBY1* gene was obtained using polymerase chain reaction containing 0.15 μg of genomic DNA, 1 unit of KOD FX neo (Toyobo, Osaka, Japan), 0.4 mM of each deoxynucleotide (dNTP), and 0.3 μM of each oligo DNA primer (5’-CAGTGTGCTGGAATTATGGCGCGCAGGGCGTCG-3’ and 5’-GATATCTGCAGAATTTCAGGCA TTCTTGTACAGCGGGAGC-3’) (Fasmac, Kanagawa, Japan) in 50 μL with incubation at 94 °C for 2 min and then 30 cycles of 98 °C for 10 s, 70 °C for 30 s, and 68 °C for 2 min. The *crtIBY1* gene was ligated to the EcoRI-digested vector pYES2 (Thermo Fisher Scientific, Waltham, MA, USA) using the In-fusion HD system (Clontech Laboratories, Mountain View, CA, USA). Stellar competent *Escherichia coli* cells (Clontech Laboratories) were transformed using a heat shock method. Resultant pYES2-*crtIBY* and pYES2 plasmids were introduced individually to *Saccharomyces cerevisiae* INVSc1 (Thermo Fisher Scientific) using the polyethylene glycol/lithium acetate method. Transformants complementing uracil requirement were isolated and cultivated in uracil-defective minimum medium (0.67% yeast nitrogen base without amino acids (Difco Laboratories, Detroit, MI, USA), 0.19% yeast synthetic drop out medium without uracil (Thermo Fisher Scientific), 4% d-raffinose (Thermo Fisher Scientific) at 28 °C for 6 h. The *crtIBY1* gene was expressed by addition of galactose (Thermo Fisher Scientific) to a final concentration of 2%, followed by further incubation for 16 h. For carotenoid analysis, cells from a 50-mL culture were collected by centrifugation (3500 rpm, 10 min, 4 °C), washed with water, and freeze-dried. Dried cells were crushed with glass beads (Wako Pure Chemical, Osaka, Japan) in 1 mL of chloroform/methanol (2:1, *v*/*v*) using a beads crusher (µT-12, Taitec, Saitama, Japan) at 2500 rpm for 120 s. The extract was recovered in the chloroform phase by centrifugation (3500 rpm, 10 min, 4 °C) of the crushed cells and evaporated under nitrogen gas spray. Carotenoids were dissolved with acetone/methanol (7:3, *v*/*v*) and analyzed on a 1260 Infinity HPLC system (Agilent Technologies, Santa Clara, CA, USA) equipped with a carotenoid separation column (S-5, 4.6 × 250 mm; YMC, Kyoto, Japan) and a photo diode array (Agilent Technologies) at 1 mL/min with acetonitrile/dichloromethane/methanol (7:2:1, *v*/*v*). Liquid chromatography-mass spectrometric (LC-MS) analysis was performed using LTQ Orbitrap XL (Thermo Fisher Scientific) equipped with a liquid chromatography (LC) column as mentioned above. We did not detect any oxidative decomposition products or other modified compounds.

## 3. Results and Discussion

### 3.1. Whole Genome Sequencing

Roche 454 WGS paired-end sequencing (661 bp on average read-length) yielded 2.9 Gb of nucleotide sequence data (Supplementary Table S1). In addition, Illumina Miseq paired-end sequencing (249 bp on average read-length, ×2) yielded 1.4 Gb of nucleotides, and GAIIx mate-pair sequencing (148 bp on average read-length, 3-kb library) yielded a total of 4.6 Gb. In total, 8.9 Gb sequence data were obtained.

The first sequence assembly yielded 2038 contigs with an N50 = 71.2 kb, and a total contig length of 76.5 Mb (Table S1). Subsequent scaffolding decreased the number of scaffolds to 512, N50 = 366.9 kb, and the total length of scaffolds was 76.7 Mb. On the other hand, k-mer analyses estimated the genome size of *Aurantiochytrium* sp. strain KH105 to be approximately 97 Mb (ranging from 94.7 to 104.1 Mb) (Supplementary Figure S1). This suggests that approximately 21% of the genome is likely repetitive or unassembled sequences. The original 8.9 Gb sequence data corresponded to approximately a 91.8-fold coverage of the genome. GC content was estimated from the assembled genome sequences to be 54.4% (Table 1; Supplementary Figure S2). This value is comparable to those of genomes for the Thraustochytrids, *Aurantiochytrium* sp. FCC1311 (56.8%) [15] and *Schizochytrium* sp. CCTCC M209059 (56.6%) [12], although the percentage is higher in *Aurantiochytrium* sp. T66 (62.8%) [13] and *Thraustochytrium* sp. ATCC 26185 (63.0%) [14] (Table 1). The GC content is also comparable for the oomycetes, *Phytophthora infestans* (51.0%), *Phytophthora sojoe* (54.4%), and *Phytophthora romarum* (54.4%) [35].

### 3.2. Gene Modeling

Gene models were constructed by combining RNA-seq sequence data (113.6 Gb of paired-end reads from the Illumina GAIIx) (Table S2) and other queries. There were 19,756 complete gene models with both start and stop codons (Table 1). Approximately 80% of them (15,800) were supported by RNA-seq analyses. At this modeling stage, the average gene length was 13,061 bp. The number of exons per gene was 4.7, and the average exon length was 513 bp, suggesting that an average gene consists of exons 2411-bp in length. This means that, on average, each gene contains an intron of nearly 10,565 bp. Here, we compared the gene content of *Aurantiochytrium* sp. strain KH105 to those of four other Thraustochytrid species (Table 1). The genome size of *Aurantiochytrium* sp. strain KH105 was almost double that of the other Thraustochytrid species. This suggests that the *Aurantiochytrium* sp. strain KH105 is diploid (see below).

The *Aurantiochytrium* sp. KH105 genome browser is accessible at Genome Projects, OIST Marine Genomics Unit website (http://marinegenomics.oist.jp/aurantiochytrium_sp_kh105, Okinawa Institute of Science and Technology, Okinawa, Japan).

To determine the ploidy level of the genome, we analyzed a set of 248 core eukaryotic genes (CEGs), which are highly conserved and are present in low copy numbers in higher eukaryotes [37]. For example, the yeasts, *Saccharomyces cerevisiae* and *Schizosaccharomyces pombe*, both haploid strains, have an average of 1.10 and 1.11 orthologs per CEG in their genomes, respectively (Supplementary Table S3), indicating that they are haploid. A similar analysis of genomes of *Phytophthora infestans* [38], *Phytophthora ramorum,* and *Phytophthora sojae* [39] showed that their average numbers were 1.25, 1.18, and 1.22, respectively. In contrast, the number of *Auranthiochytrium* sp. KH105 was 2.24, almost double those of the yeasts and *Phytophthora*. In addition, 95.6% of the 248 CEGs in the *Auranthiochytrium* sp. KH105 genome have two or more orthologs. Given the properties of CEGs, it is highly like that *Auranthiochytrium* sp. KH105 is diploid.

### 3.3. Carotenogenic Genes

**Figure 1:**
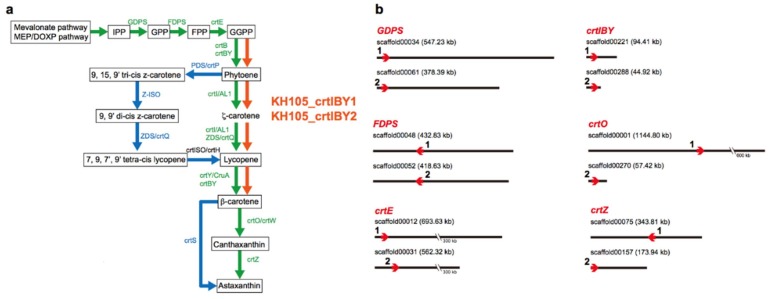
Genes encoding carotenoid biosynthetic enzymes of *Aurantiochytrium* sp. KH105. (**a**) The biosynthetic pathway for astaxanthin includes eight major enzymes, including geranyl diphosphate synthase (GDPS), farnesyl diphosphate synthase (FDPS), geranylgeranyl diphosphate synthase (CrtE), phytoene synthase (CrtB), phytoene desaturase (CrtI), lycopene cyclase (CrtY), β-carotene ketorase (CrtO), and β-carotene hydroxylase (CrtZ). Green lines indicate common pathways shared by many prokaryotes and eukaryotes, while blue lines indicate other pathways [40,41,42,43,44,45,46,47,48]. In the genome of *Aurantiochytrium* sp. KH105, three genes for *crtB*, *crtI*, and *crtY* are consecutively fused into a single, gene, *crtIBY*. The fused gene likely promotes the process from geranylgeranyl pyrophosphate (GGPP) to β-carotene; (**b**) Two copies of *GDPS*, *FDPS*, *crtE*, *crtIBY*, *crtO,* and *crtZ* were found in different scaffolds. The numbers and lengths (kb) of scaffolds are shown, and directions of the genes are indicated by arrows.

A survey of the *Aurantiochytrium* genome for enzymes involved in carotenoid biosynthesis, using sequences from other eukaryotes and prokaryotes as queries, revealed 16 and 7 enzymatic genes involved in pathways for glycolysis and mevalonate biosynthesis, respectively (Supplementary Table S4). In addition, we characterized three genes from the isopentenyl pyrophosphate (*IPP*) to geranylgeranyl pyrophosphate (*GGPP*) pathway, including geranyl diphosphate synthase (*GDPS*), farnesyl diphosphate synthase (*FDPS*), and geranylgeranyl diphosphate synthase (*crtE*), and five genes from *GGPP* to astaxanthin pathway, including phytoene synthase (*crtB*), phytoene desaturase (*crtI*), lycopene cyclase (*crtY*), β-carotene ketolase (*crtO*), and β-carotene hydroxylase (*crtZ*) (Figure 1a; Supplementary Table S4). All eight of these genes appear duplicated. That is, two copies of *GDPS* were found in scaffolds 00034 and 00061, those for *FDPS* in scaffolds 00048 and 00052, those for *crtE* in scaffolds 00012 and 00031, those for *crtB*, *crtI,* and *crtY* in 00221 and 00288, those for *crtO* in 00001 and 00270, and those for *crtZ* in 00075 and 00157 (Figure 1b). RNA-seq analysis also confirmed the duplication of these genes.

### 3.4. Structure and Function of crtIBY Gene

**Figure 2:**
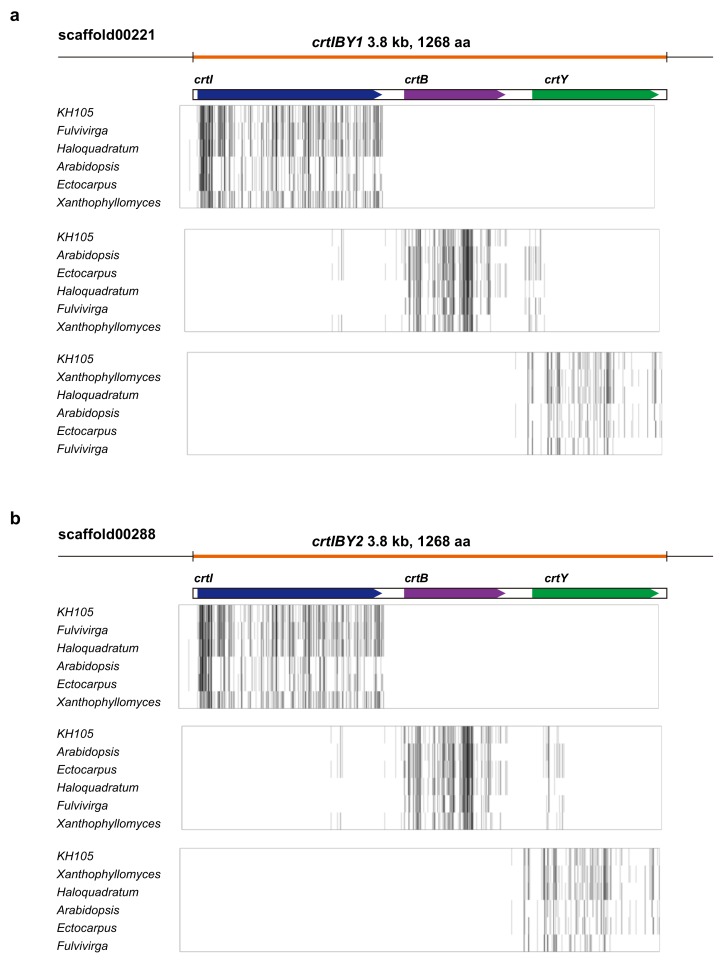
Composition of *crtIBY1* (**a**) and *crtIBY2* (**b**), and their amino acid alignments with genes of other organisms. Darker lines indicate higher amino acid similarity. GenBank/DDBJ accession numbers are indicated in Supplementary Table S5.

In addition, we found that *crtB*, *crt I,* and *crtY* were clustered in scaffolds 00221 (tentatively called *crtIBY1*) and 00288 (*crtIBY2*), and that the gene order in both clusters was *I-B*-*Y* (Figure 2). Both expanded approximately 3.8 kb in the scaffolds and were expected to encode proteins of 1,268 amino acids. Molecular phylogeny indicated an affinity of KH105 CrtI (Figures S3 and S4), CrtB (Supplementary Figures S5 and S6), and CrtY (Figures S7 and S8) to archaean homologs. Messenger RNAs (mRNAs) transcribed by *crtIBY1* and *crtIBY2* (for example, base positions 106 to 3807 of *crtIBY1* (Figure 2) had an open reading frame (ORF) without stop codons. This suggests that the mRNAs are translated to produce single polypeptides of 1268 amino acids.

Therefore, not only are *crtB*, *crtI,* and *crtY* clustered in the genome, but they are also fused into a single gene. Although there are several reports that show a cluster of two genes encoding carotenoid biosynthetic enzymes, for example, *carRA* in *Phycomyces blakesleeanus* [39] and *crtYB* in *Xanthophyllomyces dendrorhous* [49], this finding of a three-gene fusion appears novel. Since the biosynthetic process from GGPP to β-carotene is usually regulated by four independent enzymatic reactions catalyzed by CrtB, CrtI, and CrtY (green lines in Figure 1a), this gene fusion suggests that *Aurantiochytrium* has managed to increase efficiency by performing all three reactions with a single, multifunctional enzyme (Figure 1a). In addition, we have been unable to detect lycopene, which is an intermediate product of the pathway when the process is accomplished by four separate enzymatic reactions.

**Figure 3:**
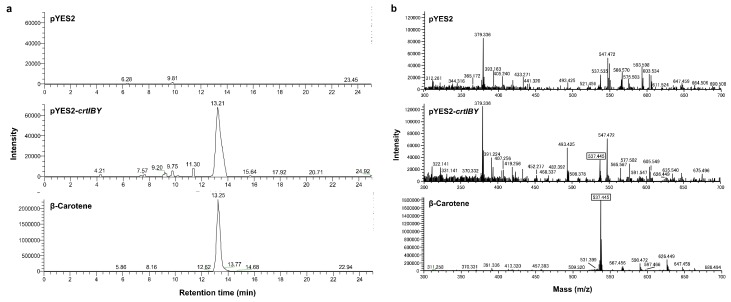
Liquid chromatography–mass spectrometric analyses of carotenoids generated by yeasts expressing *crtIBY* from *Aurantiochytrium* sp. KH105. (**a**) Total ion chromatograms of carotenoids extracted from yeast cells carrying pYES2 or pYES2-*crtIBY* and the β-carotene standard. An ion signal was selected within the molecular weight range of 537.44–537.45; (**b**) Mass spectrometry of carotenoids extracted from yeast cells carrying pYES2 or pYES2-*crtIBY* and the β-carotene standard at the retention times of 13.35, 13.21, and 13.22 min, respectively.

To confirm the function of *crtIBY*, the gene was heterologously expressed in yeast *Saccharomyces cerevisiae* INVSc1, which possesses GGPP synthase (BTS1), but lacks carotenoid biosynthetic genes, *crtB*, *crtI,* and *crtY*. Liquid chromatography–mass spectrometric analysis of carotenoids produced by the *crtIBY1*-expressing yeast identified a newly generated compound as β-carotene, based upon its mass profile, which is similar to that of authentic β-carotene (Figure 3). This indicates that the *crtIBY* gene encodes β-carotene synthase. The fact that levels of β-carotene increased in BTS1-overexpressing cells suggests that GGPP is the substrate of CrtIBY.

### 3.5. Gene Expression Profile

**Figure 4:**
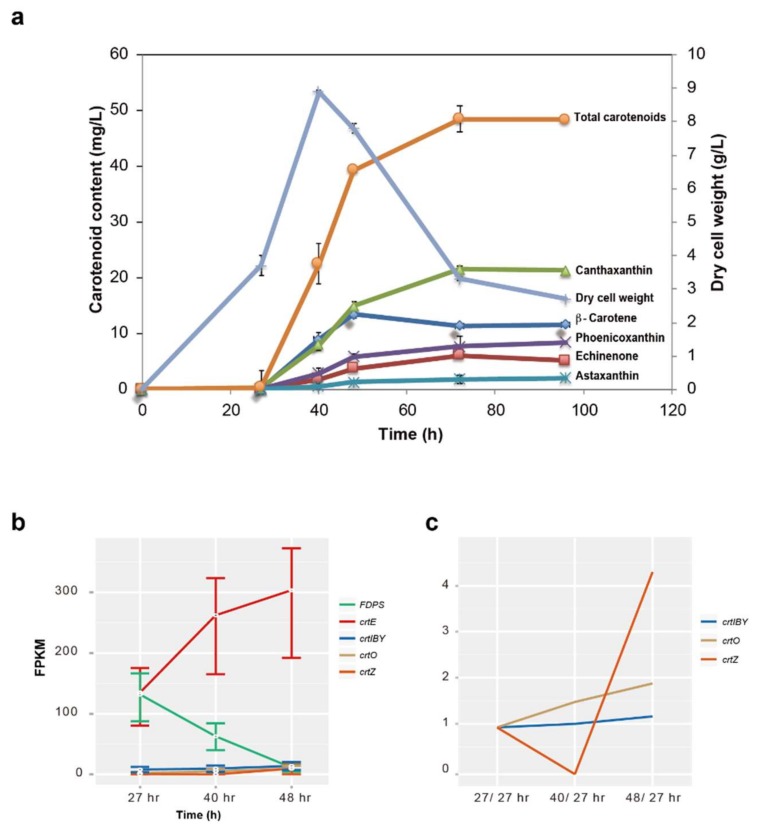
Differential expression profiles of genes for carotenoid synthetic enzymes of *Aurantiochytrium* sp. KH105. (**a**) Time course of total carotenoid synthesis (left scale) by cells incubated at 28 °C in relation to cell number (right scale; DCW, dry cell weight); (**b**) Differential gene expression profile analysis showing changes during cultivation time from 27 h (mid-log phase), 40 h (late-log to stationary phase), and 48 h (starvation phase) in the concentration of messenger RNAs (mRNAs) of *FDPS*, *crtE*, *crtIBY1*, *crtO,* and *crtZ*. Three independent measurements were carried out. Fragments per kilobase of transcript per million mapped reads (FPKM), fragments per kilobase of exon per million reads mapped; (**c**) Comparison of the concentration of mRNAs of genes for *crtIBY1*, *crtO* and *crtZ*, between 27/27 h, 40/27 h and 48/27 h, respectively.

Carotenoid biosynthesis of *Aurantiochytrium* sp. KH105 becomes evident after 27 h of culture at 28 °C (mid-log phase of culture; Figure 4a), and carotenoid production plateaus after 72 h (starvation phase). Amounts of β-carotenes, canthaxanthin, and astaxanthin, plateaued at ≈48 h (stationary phase), 72 h, and 96 h, respectively, after initiation of incubation. We thought that these changes might reflect enzymatic gene expression; therefore, we performed a large-scale, gene expression profile analysis of the seven genes involved in carotenoid synthesis. This analysis showed that the concentration of *FDPS* mRNA decreased after 27 h of incubation (Figure 4b), while the concentrations of *crtE*, *crtIBY*, *crtO,* and *crtZ* mRNAs steadily increased from 27–48 h of incubation.

Major lipid biosynthetic pathways in *Aurantiochytrium* include those for polyunsaturated fatty acids (DHA and DPA) from acetyl-CoA, squalene, and sterols from farnesyl pyrophosphate (FPP), and xanthophylls from FPP/GGPP (Supplementary Figure S9). As mentioned above, in strain KH105, increased expression activity of *crtE* was evident, but overall expression levels of *crtIBY*, *crtO,* and *crtZ* appeared lower compared to that of *crtE.* Therefore, a key enzymatic reaction to synthesize xanthophylls is the CrtIBY-mediated reaction to produce β-carotene from GGPP. Although future studies should explore regulatory mechanisms of *crtIBY* gene expression in relation to *crtE* expression, genetic manipulation of the expression of these genes seems to be a key event that has allowed *Aurantiochytrium* to produce carotenoids more efficiently.

Since differential gene expression profile analysis showed fewer changes in the concentration of mRNAs for *crtIBY*, *crtO,* and *crtZ*, we further compared mRNA levels of the three genes between growth phases. Expression levels of *crtO* and *crtZ* increased from 27 (mid-log phase) to 40 h (late-log to stationary phase), and from 27 to 48 h (starvation phase), respectively (Figure 4c). Up-regulation of *crtO* and *crtZ* expression appeared to correspond well to increased concentrations of canthaxanthin and astaxanthin (Figure 4a,c). This suggests that genetic changes that up-regulate *crtO* and *crtZ* expression do increase carotenoid production. However, since overall levels of *crtO* and *crtZ* expression appear high during log phase compared to starvation phase, this explanation may be too simplistic. In contrast, the expression level of *crtIBY* appears lower than those of other enzymatic genes. Therefore, a plausible way to increase carotenoid production is to induce higher *crtIBY* expression during log, stationary, and starvation phases.

### 3.6. Occurrence of the crtIBY Gene in other Thraustochytrid Species

**Figure 5:**
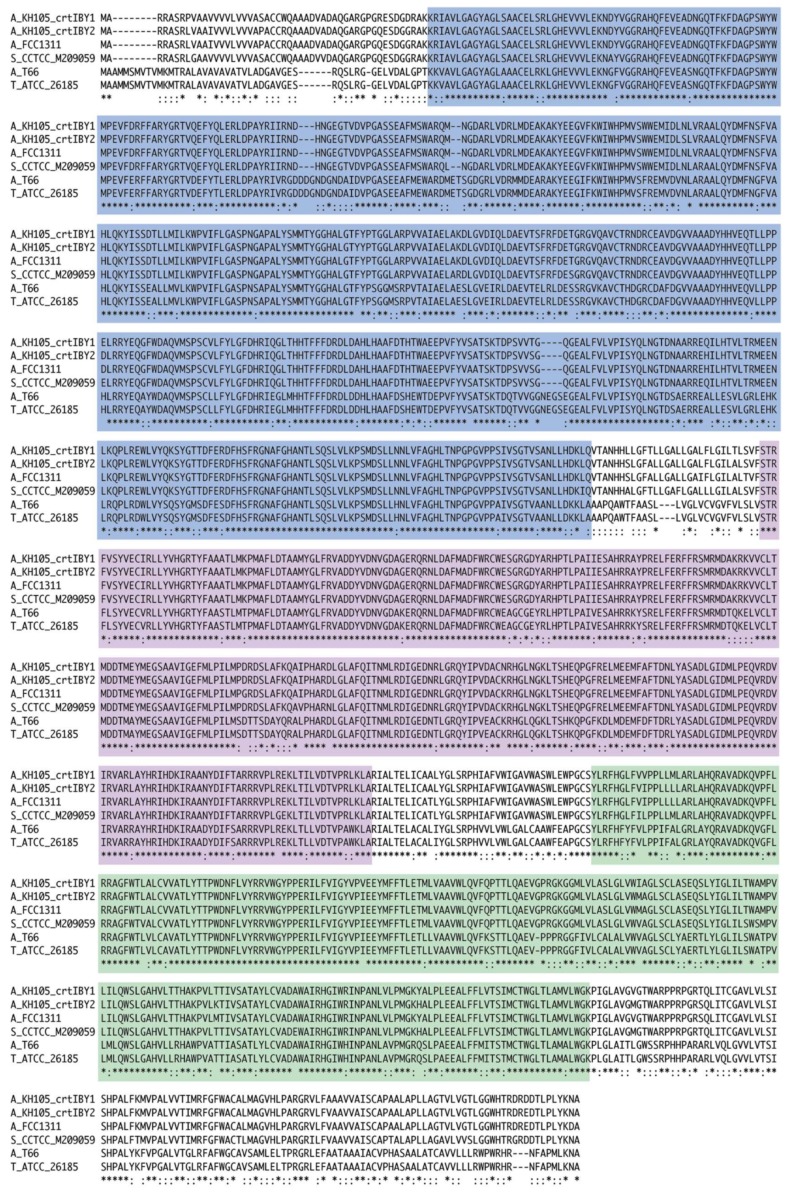
Alignment of amino acid sequences of possible trifunctional β-carotenase synthases, CrtIBY. Sequences of CrtIBY1 and CrtIBY2 of *Aurantiochytrium* sp. KH105 are compared with those of *Aurantiochytrium* sp. FCC1311, *Schizochytrium* sp. CCTCC M209059, *Aurantiochytrium* sp. T66, and *Thraustochytrium* sp. ATCC 26185. Blue indicates CrtI, purple, CrtB, and green, CrtY. Asterisks indicate identical amino acid residues; colons denote difference between the upper three and the lower two species; hyphens indicate missing residues.

As described above, the genome of *Aurantiochytrium sp*. strain KH105 contains a fused gene, *crtIBY*, and the encoded protein likely acts as a trifunctional β-carotene synthase. Because genomes of other Thraustochytrid species have been sequenced (Table 1), we examined whether *crtIBY* is specific to *Aurantiochytrium* sp. 105 or whether it is also a feature of other Thraustochytrids. As a query of KH105 *crtI*, *crtB, or crtY* nucleotide sequences, we BLASTed genome sequences of the other four species. We found that all four Thraustochytrids possess a protein very similar to KH105 CrtIBY (Figure 5). No stop codons were found in the genomic region that includes the sequences. Especially, amino acid sequences of CrtIBY were almost completely identical among *Aurantiochytrium* sp. strain KH105 and *Aurantiochytrium* sp. FCC1311 and *Schizochytrium* sp. CCTCC M209059 (Figure 5). On the other hand, in CrtI of *Aurantiochytrium* sp. T66 and *Thraustochytrium* sp. ATCC 26185, there were three insertions consisting of two, three, or four amino acids. In addition, there were many amino acid substitutions in these two species, compared with the other three (Figure 5). Therefore, it is highly likely that a fused gene *crtIBY* is common to all Thraustochytrid species, although its function should be examined in each.

## 4. Conclusions

The decoded genome of *Aurantiochytrium* sp. strain KH105 demonstrated at least two genomic alterations associated with efficient production of carotenoids, gene duplication, and gene fusion. The fusion gene, *crtIBY*, looks common in all Thraustochytrid species. In addition, a large number of differential expression profiles of genes involved in carotenoid biosynthesis suggest key genetic manipulations that lead to more efficient production of carotenoids in this strain.

## Figures and Tables

**Table 1:** Genome assembly and annotation statistics for *Aurantiochytrium* sp. KH105 and their comparison with those of other Thraustochytrid species.

	*Aurantiochytrium* sp. KH105 ^(1)^	*Aurantiochytrium* sp. T66 ^(2)^	*Aurantiochytrium* sp. FCC1311 ^(3)^	*Schizochytrium* sp. CCTCC M209059 ^(4)^	*Thraustochytrium* sp. ATCC 26185 ^(5)^
Genome					
Estimated genome size (Mb)	95	43	39	39	39
Coverage (fold)	8	303	40	55	62
Number of scaffolds	1810	1847	2232	322	2250 (10,764)
N50 scaffold length (kb)	463	1342.8	236.6	595.8	239.0 (2139)
Total scaffold length (Mb)	86.0	43.4	38.9	39.1	38.6 (18.1)
Number of contigs	5577	6833	4504	1608	8130 (10,768)
N50 contig length (kb)	105.7	12,952	22,474	52	(2139)
Total contig length (Mb)	78	38.3	38.7	38.3	18
G+C content (%)	54.4	62.8	56.8	56.6	63.0 (56.0)
Genes					
Number of gene models	16,988	11,683	11,853	9142	10,797
Fused CrtIBY	+	+	+	+	+

^(1)^ The present study is accessible at Genome Projects, OIST Marine Genomics Unit website (http://marinegenomics.oist.jp/aurantiochytrium_sp_kh105, Okinawa Institute of Science and Technology, Okinawa, Japan); ^(2)^ Liu et al. [13]; ^(3)^ Sediki et al. [15,36]; ^(4)^ Ji et al. [12]; ^(5)^ Zhao et al. [14]. The number in parenthesis comes from the data bank.

## Supplementary Materials

Supplementary materials can be found at http://www.mdpi.com/2073-4425/9/4/200/s1.
