# Supplementary material for: A Possible Trifunctional β-Carotene Synthase Gene Identified in the Draft Genome of Aurantiochytrium sp. Strain KH105
Source: Genes (Basel). 2018 Apr 9;9(4):200. doi: 10.3390/genes9040200 (PMC5924542; doi:10.3390/genes9040200)
Supplement: Supplementary file 1 [file genes-09-00200-s001.zip › Supplement/Fig. S1.pdf]

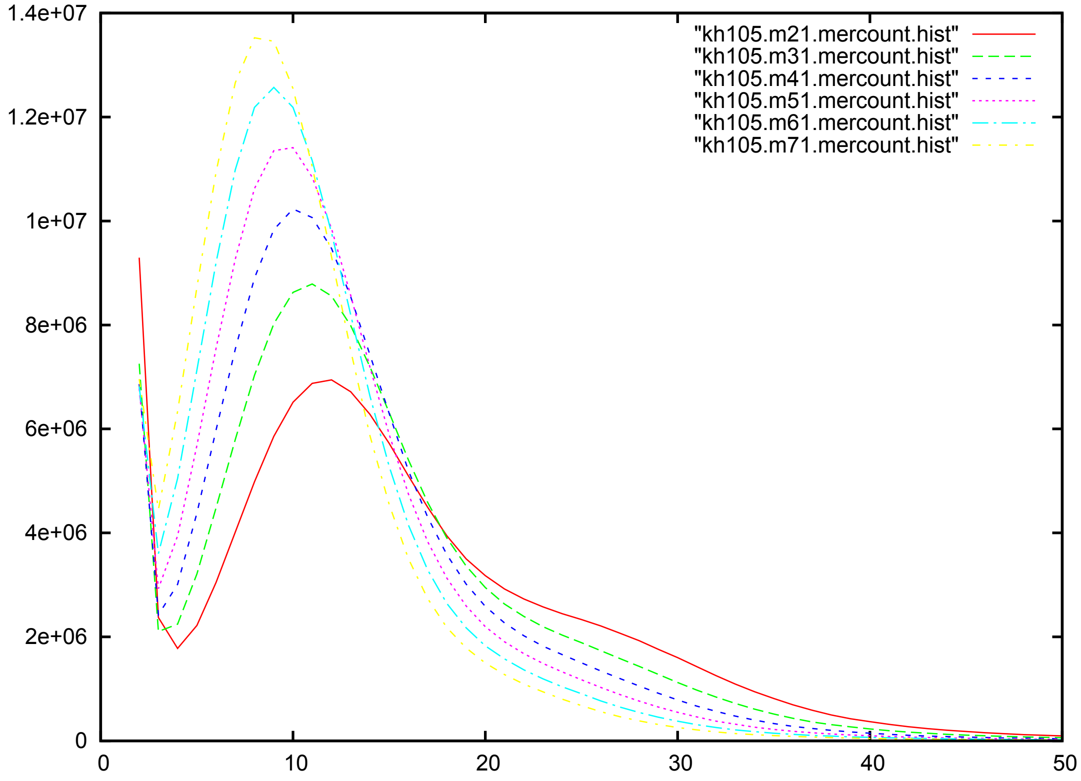

Supplementary Figure S1: A k-mer analysis of quality-trimmed Illumina reads (113.6 Gbp). The frequency distributions of distinct k-mers (21, 31, 41, 51, 61, and 71) are shown. Peaks appear around 9-11 K-mer coverage. This value suggests that the *Aurantiochytrium* haploid genome is 94-104 Mbp.
