# Supplementary material for: A Possible Trifunctional β-Carotene Synthase Gene Identified in the Draft Genome of Aurantiochytrium sp. Strain KH105
Source: Genes (Basel). 2018 Apr 9;9(4):200. doi: 10.3390/genes9040200 (PMC5924542; doi:10.3390/genes9040200)
Supplement: Supplementary file 1 [file genes-09-00200-s001.zip › Supplement/Fig. S2.pdf]

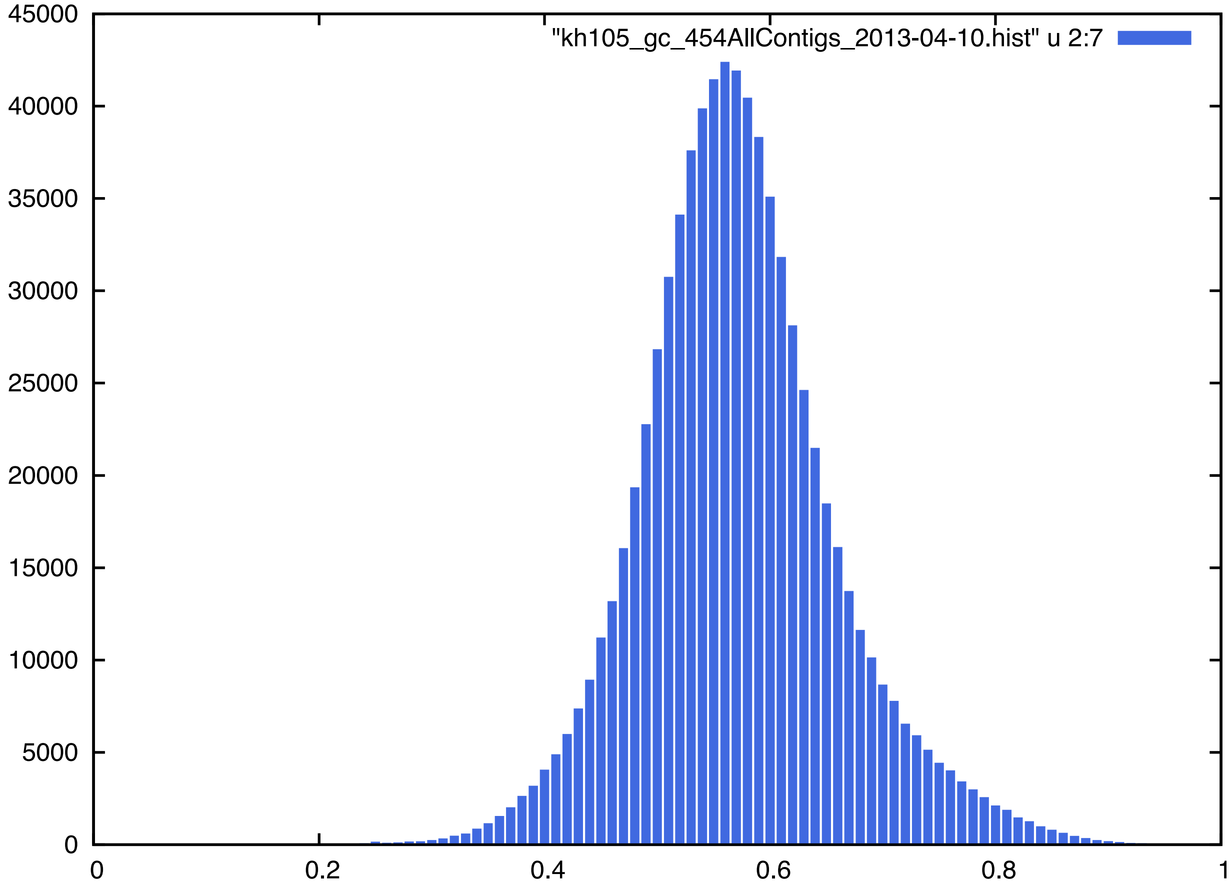

Supplementary Figure S2: GC content within sequences of the *Aurantiochytrium* sp. KH105 genome. The vertical axis shows the number of each region 50 bp window while the horizontal axis indicates GC content (%). The peak indicates that the GC content is 55%.
