# Supplementary material for: A Possible Trifunctional β-Carotene Synthase Gene Identified in the Draft Genome of Aurantiochytrium sp. Strain KH105
Source: Genes (Basel). 2018 Apr 9;9(4):200. doi: 10.3390/genes9040200 (PMC5924542; doi:10.3390/genes9040200)
Supplement: Supplementary file 1 [file genes-09-00200-s001.zip › Supplement/Fig. S3.pdf]

```
KH105_crtIBY1        1 ----------------------------------------------MARRASRPVAAVVV
KH105_crtIBY2        1 ----------------------------------------------MARRASRLVAAIVV
Brevundimonas        1 ------------------------------------------------------------
Xanthophyllomyc      1 ------------------------------------------------------------
Arabidopsis          1 MVVFGN---VSAANLPYQNGFLEALSSGGCELMGHSFRVPTSQALKTRTRRRSTAGPLQV
Haematococcus        1 -----------------MHTTMRGQASGSGCTSGR-QTRGHWSRRSVRERG-----ALRV
Thalassiosira        1 ----------------------------------------MLPHTTVHGVVALATLLLNA
Ectocarpus           1 MVGAGGGAFLALALAGKTISAFVLPPATSFTGVGLSGSARQSAIRRNGVSSMIFDFPAPG
Chlamydomonas        1 -----------------MQTQVKPSSSRQANLVAKGASCPRVAVRRVAGRR-----ALEV
Synechococcus        1 ------------------------------------------------------------
Haliscomenobact      1 ------------------------------------------------------------
Saprospira           1 ------------------------------------------------------------
Fulvivirga           1 ------------------------------------------------------------
Pedobacter           1 ------------------------------------------------------------
Cyclobacterium       1 ------------------------------------------------------------
Haloquadratum        1 ------------------------------------------------------------
Natrinema            1 ------------------------------------------------------------

KH105_crtIBY1       15 VLVVVASACCWQAAADVADAQGARGPGRESDGDRAKKRIAVLGAGYAGLSAACELSRLGH
KH105_crtIBY2       15 VLVVVAPACCRQAAADVADAQGARGPGQESDGGRAKKRIAVLGAGYAGLSAACELSRLGH
Brevundimonas        1 ------------------------------------MRAAVIGSGFGGLSLAIRLQTAGI
Xanthophyllomyc      1 ---------------------------MGKEQDQDKPTAIIVGCGIGGIATAARLAKEGF
Arabidopsis         58 VCVDIPRPELENT-VNFLEAASLSASFRSAPRPAKPLKVVIAGAGLAGLSTAKYLADAGH
Haematococcus       38 VAKDYPTPDFQSS-DTYQEALSLSTKLRNAPRPAKPLRVVIAGAGLAGLSAAKYLADAGH
Thalassiosira       21 FVLVDSFAPSPRCSHRYHISSAASTTLHATTTPHSEYDAIIVGSGIGGLSAAALLSHYGY
Ectocarpus          61 ERKDYPRPPLEETSENQRESAAFSQAFLDMPRPERPLKVAVIGAGLAGLSCAKYLSDAGH
Chlamydomonas       39 VARDYPRPAFETA-ETFQEAKALSSKLKDAPRPAKPLKVVIAGAGLAGLSAAKYLSDAGH
Synechococcus        1 ------------------------------------MRVAIAGAGLAGLSCAKYLADAGH
Haliscomenobact      1 --------------------------MS--------KKVVVIGAGFAGLAAACCLAKAGY
Saprospira           1 --------------------------MS--------KKVIVIGSGFAGLSAACHLAKAGY
Fulvivirga           1 --------------------------MSK-------KKIAVIGAGFAGLSAACCLADAGF
Pedobacter           1 --------------------------MPVNSDHKIVPKVAVIGSGFAGLSAAAYLAKAGC
Cyclobacterium       1 --------------------------MKP-------KEVVVIGSGFSGLSTATHLAAAGY
Haloquadratum        1 --------------------MNTVSDLSDVDG----SSVVVIGGGFGGLSTACYLSDAGA
Natrinema            1 --------------------------MQSLAG----ESVVVIGSGIGGLSTACYLADAGA

KH105_crtIBY1       75 EVVVLEKNDYVGGRAHQFEVEADNGQTFKFDAGPSWYWMPEVFDRFFARYGRT-VQEFYQ
KH105_crtIBY2       75 EVVVLEKNDYVGGRAHQFEVEADNGQTFKFDAGPSWYWMPEVFDRFFARYGRT-VQEFYQ
Brevundimonas       25 QTTVFEARDLPGGRAYVYKDK-----GYTFDAGPTVITDPSALEELFEGAGRK-LSDYVE
Xanthophyllomyc     34 QVTVFEKNDYSGGRCSLIERD-----GYRFDQGPSLLLLPDLFKQTFEDLGEK-MEDWVD
Arabidopsis        117 KPLLLEARDVLGGKIAAWKDED----GDWYETGLHIFFG--AYPNVQNLFGELGINDRLQ
Haematococcus       97 HPVVLEGRDVLGGKVAAWKDED----GDWYETGLHIFFG--AYPNIQNLFKELGIQDRLQ
Thalassiosira       81 SVAVFEAHSTPGGAAHGYTVNAKDVGPLTFDTGPSFFSGLNSNYPAKSSNPLRSILDIID
Ectocarpus         121 KPVVLEARDVLGGKVAAWQDKD----GDWVETGLHIFFG--AYPNVNQMFTELGIRDRLQ
Chlamydomonas       98 HPIVLEGRDVLGGKVAAWKDED----GDWYETGLHIFFG--AYPNMMNVFKELNIEERLQ
Synechococcus       25 TPIVVEARDVLGGKVAAWKDED----GDWYETGLHIFFG--AYPNMLQLFKELNIEDRLQ
Haliscomenobact     27 EVTVLEKNSVPGGRARQYSAD-----GFVFDMGPSWYWMPDVFEQFFAHFGKK-TSDYYQ
Saprospira          27 SVQLLEKNDCAGGRARKMEVQ-----GYHFDMGPSWYWMPEVFEQFFEQFGKK-VSDYYE
Fulvivirga          28 NVTVFEKNASPGGRARKFDVQ-----GFTFDMGPSWYWMPDVFERFFNRFGKS-TSDYYT
Pedobacter          35 SVDVYEKNSSIGGRARQLKTEN----GYLFDMGPSWYWMPEVFERFFNDFGYK-ASDFYE
Cyclobacterium      28 KVTVLEKNEVLGGRARKFNSA-----GFTFDMGPSWYWMPDVFDRYFSNFGKK-TSDYYD
Haloquadratum       37 DVTVLEKNEQLGGRASRLEVD-----GFKFDMGPSWYLMPDVFERFFNYFDRE-PSEYYE
Natrinema           31 DVRVIEKNEQLGGRASRLEKD-----GFTFDMGPSWYLMPDVFERFFADFDRT-PGDYYD
```

Supplementary Figure S3: Alignment of CrtI1 and CrtI2 amino acids within CrtKH105_CrtIBY1 and CrtKH105_CrtIBY2 with those of other eukaryotes.

```
KH105_crtIBY1     134 LERLDPAYRIIRNDHNGEGTVDVPGASSEAFMSWARQM-----------NGDARLVDRLM
KH105_crtIBY2     134 LERLDPAYRIIRNDHNGEGTVDVPGASSEAFMSWARQM-----------NGDARLVDRLM
Brevundimonas      79 LLPVAPFYRLC---WEDGDVFDYVNGQDELDRQIVARN-----------PADKEGYRRFL
Xanthophyllomyc    88 LIKCEPNYVCH---FHDEETFTLSTDMALLKREVERFEG----------KDGFDRFLSFI
Arabidopsis       171 WKEHSMIFAMP-----SKPGEFSRFDFPDVLPAPLNGI-----------WAILRNNEMLT
Haematococcus     151 WKEHSMIFAMP-----DAPGEFSRFDFPE-LPAPWNGI-----------IAILRNNQMLS
Thalassiosira     141 EKVECIPYTTFG-LMFPEGVFVHSSNFGKEGSTVEAVSGSNGVQEWASLMKSMDPLAQAV
Ectocarpus        175 WKSHSMIFAMPGQQTADGFQRFSRFEFPALLPAPLNGL-----------VAILLNNEMLT
Chlamydomonas     152 WKEHSMIFAMP-----DSPGEFSRFDFPD-IPAPFNGV-----------FAILRNNQMLT
Synechococcus      79 WKSHSMIFNQQ-----DEPGTYSRFDFPD-LPAPVNGV-----------AAILGNNDMLT
Haliscomenobact    81 LQRLDPSYSIF---FGEDEVMQVPANMEELFAMFERYE-----------PGSSRNLKKFL
Saprospira         81 LKRLDPAYKIV---FGEGEELAVPADMQALKELFESYE-----------PGSSAKLQQFL
Fulvivirga         82 LQRLNPSYKVF---FKDE-SIDIPAALQDIYALFDTIE-----------PGSSTNLRQFL
Pedobacter         90 LELLNPGFSIV---FGMEDVMNVPADFNELCALFESLE-----------TGSAAKLKQFL
Cyclobacterium     82 LIRLDPSYSVI---FGQEDVMEIPANLDQFKLALEKLE-----------PGVSEKLDDFL
Haloquadratum      91 LTRLDPHYRIF---FKDGDRVEMVPDREQNREVFESYE-----------PGAGDRFDEYL
Natrinema          85 LEHLDPHYRIF---FKDGDRVDITPDLERTKAVFEEYE-----------SGAGEALERYL

KH105_crtIBY1     183 DEAKAKYEEGVFKWIWHPMVSWWEMIDLNLVRAALQYDMFNSFV--AHLQKYISSDTLLM
KH105_crtIBY2     183 DEAKAKYEEGVFKWIWHPMVSWWEMIDLSLVRAALQYDMFNSFV--AHLQKYISSDTLLM
Brevundimonas     125 AYSQDLLKEGYLKLGAVPFLDFAS--MVKAAPELMRLQAWRSVY--DKVAGYIQDEHLRQ
Xanthophyllomyc   135 QEAHRHYELAVVHVLQKNFPGFAAFLRLQFIGQILALHPFESIW--TRVCRYFKTDRLRR
Arabidopsis       215 WPEKIKFAIGLLPAMVGGQAYVEAQDGLSVKEWMEKQGVPERVT--DEVFIAMS-----K
Haematococcus     194 WPEKIRFAIGLLPAIIFGQRYCEEQDELTVTEWMRKQGVPDRVN--EEVFIAMA-----K
Thalassiosira     200 DAMPTLALRADLGLLASTSQFLPNFAKLNPLQNLKLTKPFSNIINEAGVKDTFIRNWLDV
Ectocarpus        224 FPEKIQFGIGLLPAILFGQKYVEECDSLTVSQWMKKQGVPDRVN--DEVFIAMA-----K
Chlamydomonas     195 WPEKIQFAIGLLPAIIFGQKYVEEQDHLSVTQWMRQQGVPDRVN--EEVFIAMA-----K
Synechococcus     122 WPEKISFGLGLVPAMLRGQGYVEECDKYSWTEWLRVHNIPERVN--DEVFLAMS-----K
Haliscomenobact   127 EEAKYKYEVGMADFVNKPSHSILEFADLRLLKSMFRLQMFTSMS--THVRQLFNNRQLIE
Saprospira        127 DEAEYKYKVGMGEFVQKPSLSILEFADLRVVRALFKLQMFSSIA--THVEKLFKHPHLRE
Fulvivirga        127 EEAAYKYEVGINDLVYKPGRSVTEFLDMRLMHGIFRLHVFNSIA--NYIRKYFSHPKLIQ
Pedobacter        136 KEAEFKYDVGMGKLVYKPGLSLLEFADADLIKGMFKLQVFNSFS--KHVRQFFKHPQLIA
Cyclobacterium    128 EQAAFKYQRGINDLVHRPSVSLTEFLDLKLFRDVLKMDIFQSMS--KHIRKYFKNEKIIR
Haloquadratum     137 AKSKKNYEVGMEHFVYEDRAELSDYADWDVMKNARGLSLIGSMQ--DHVEQYFDHPKLQQ
Natrinema         131 EKSQENYEVGMKHFVYEDRERLRDYLDLDVARQARGLSLLGSMQ--GHVAGYFDHPKLQQ

KH105_crtIBY1     241 ILKWPVIFLGASPNGAPALYSMMTYGGHALGTFYPTGGLARPVVAIAELAK--DLGVDIQ
KH105_crtIBY2     241 ILKWPVIFLGASPNGAPALYSMMTYGGHALGTYYPTGGLARPVVAIAELAK--DLGVDIQ
Brevundimonas     181 AFSFHSLLVGGNPFATSSIYALIHALERRWGVWFPRGGTGALIQAMVRLFQ--DLGGEIR
Xanthophyllomyc   193 VFSFAVMYMGQSPYSAPGTYSLLQYTELTEGIWYPRGGFWQVPNTLLQIVKRNNPSAKFN
Arabidopsis       268 ALNFINPDELSMQCILIALNRFLQEKHGSKMAFLDGNPPERLCMPVVDHIR--SLGGEVQ
Haematococcus     247 ALNFINPDDLSMTVVLTALNRFLQEQHGSKMAFLDGAPPERLCQPMVDYFK--ARGGDLM
Thalassiosira     260 LCFCLSGVPSDGTITAEMAMMMGEFYDEDAIMDCPVGGASAIVDALVRGIE--KKGGKVF
Ectocarpus        277 ALNFIDPENLSMTVVLTALNRFLQETHGSKMAFLDGPPPTRLCQPMADHML--ARGGELR
Chlamydomonas     248 ALAFIDPDRLSMTVVLTALNRFLQERHGSKMAFLDGAPPERLCQPMVDHFT--ARGGELK
Synechococcus     175 ALNFIDPDEISATVVLTALNRFLQEKSGSKMAFLDGAPPERLCQPVVEHIE--SLGGEVH
Haliscomenobact   185 LLEFPVLFLGATPAKTPAMYSLMNYADMALGTWYPRGGMYKIVEAMVSLAR--ELGVDLQ
Saprospira        185 LLKFPVLFLGATPEETPAMYSLMNYADLALGTWFPMGGMYQIVAAMVSLAE--ELGVEIL
Fulvivirga        185 LLEFPILFLGAMPRKTPALYSLMNYADMALGTWYPEGGMHKIVEAMETLAL--SLGVRFE
Pedobacter        194 LMEFPVLFLGAMPEDTPALYSLMNYAGLKLGTWYPKGGFAAVIQAMKTVAE--RQGVKFH
Cyclobacterium    186 LMEFPVLFLGETAQNIPALYSLMNYADIALGTWYPKGGMHKIIEGMVTLAK--DNGVVFR
Haloquadratum     195 IMQYTLVFLGGAPTNTPALYNLMSHVDFNMGVYYPENGVGGVVDGIVELAD--ELGVTFH
Natrinema         189 IMQYTLVFLGGSPTNTPALYNLMSHVDFNLGVWYPDGGIGAVIDGIAELGA--ELGVEYD
```

```
KH105_crtIBY1    299 LDAEVTSFRFDETGRGVQAVCTRND---RCEAVDGVVAAADYHHVEQTLLPPELR-----
KH105_crtIBY2    299 LDAEVTSFRFDETGRGVQAVCTRND---RCEAVDGVVAAADYHHVEQTLLPPDLR-----
Brevundimonas    239 LNSPVERITLANG--RADGVVVGG----QALAFDMVASNADVVHTYQRLLGQEPRG----
Xanthophyllomyc  253 FNAPVSQVLLSPAKDRATGVRLESG---EEHHADVVIVNADLVYASEHLIPDDARNKIGQ
Arabidopsis      326 LNSRIKKIELNDDGTVKSFLLT-----NGSTVEGDAYVFAAPVDILKLLLPDPWK-----
Haematococcus    305 FNSRVKQIVLNDDKSVKHLALT-----NGQTVEGDLYISAMPVDIMKILMPDPWA-----
Thalassiosira    318 CNSRIDEICIENGKAVGVRLAKNYS---RIKATKGVISNLSVWDLMNSGIVDTDL-----
Ectocarpus       335 MEQRISEILLNDDKTVKGLKMQ-----DGSTVVADAYVSTMPVDILKLALPEAWR-----
Chlamydomonas    306 MNARVKDIVLNDDGSVKHYKLT-----TGEVVEGDLYMSAMPVDILKLLVPDQWK-----
Synechococcus    233 LDSPLREIKLNEDGSVAAFHIGGVKGKDSFDLTADAYVSALPVDPFKLLLPKPWK-----
Haliscomenobact  243 LNQEVKQILAPHG--LATKVITQD----HEYTADAVVGGADYHHLDQQVLAPEQR-----
Saprospira       243 LNEEVLSIEVSGK--QAKAAITKN----GRFEADIIVAGADYHHVDQKLLAPEYR-----
Fulvivirga       243 YNAAVSAIEVKEN--HAKGVVVDN----TLIEHDIIIGGADYHHIEQHLLPEKYR-----
Pedobacter       252 TDTNVDGLEVQAR--KLQALKLAN----SSEAYDGLIAAADYHHVESQLLGPAYR-----
Cyclobacterium   244 NNAEVVKIMADDG--KAKSVQLLSG---EEIAFDILVASADYHHVDKHLLEPKYS-----
Haloquadratum    253 TNAPVSEIRGREG---AFVTRTETD---AEFYSESVVSNADYRHTEMELLPPEKR-----
Natrinema        247 TDRPATEIKGRTG---GFEVETP-A---GPLQSDLVVSNADYAHTEQELLTPERR-----

KH105_crtIBY1    351 --RYEQGFWDAQVMSP------SCVLFYLGFDHRIQGLTHHTFFFDRDLDAHLHAAFDTH
KH105_crtIBY2    351 --RYEQGFWDAQVMSP------SCVLFYLGFDHRIQGLTHHTFFFDRDLDAHLHAAFDTH
Brevundimonas    289 --RKEGARLASKRHSM------SLFVIYFGLKRVHPEVRHHTVLFGPRYRELIGEIFKGP
Xanthophyllomyc  310 LGEVKRSWWADLVGGKKLKGSCSSLSFYWSMDRIVDGLGGHNIFLAEDFKGSFDTIFEEL
Arabidopsis      376 --EIPYFKKLDKLVGV------PVINVHIWFDRKLKNTYDHLLFSRSNLLSVYADMSLTC
Haematococcus    355 --SMPYFKQLNGLEGV------PVINIHIWFDRKLT-TVDHLLFSRSPLLSVYADMSTTC
Thalassiosira    370 ---FPEDFVKERKATP---ACPSFMHLHVGFQITKEELSKLQAHY------IFMNDWERG
Ectocarpus       385 --PMPYFEKLNGLNGV------PVINIHMWFDRKLT-TVDHLLFSRSPLLSVYADMSLTC
Chlamydomonas    356 --PNPYFSQLKELEGV------PVINIHIWFDRKLT-TVDHLLFSRSPLLSVYADMSTTC
Synechococcus    288 --QMEVFRKLDGLRGV------PVINLHLWFDRKLT-DIDHLLFSRSPLLSVYADMSITC
Haliscomenobact  292 --NYDAAYWDKRVMAP------SSLLFYLGVNKRLKNLHHHNLFFDEDFTQHAIEIYDDP
Saprospira       292 --NYNERYWDKRVMAP------SSLLFYLGVNKPLNNLLHHNLFFDEDFKLHAQEIYKTP
Fulvivirga       292 --KYDQKYWDKRALAP------SSLIFYLGVDKKISHLLHHNLFFDEDFEQHAQEIYEQP
Pedobacter       301 --NYSEQYWSSRVLAP------SSLIFYLGIDRKIPGLNHHTLFFDEDLNQHAQEIYKAP
Cyclobacterium   294 --NYSDKYWDSRVLAP------SSLLFYLGVNKRLKNLLHHNLFFDEPLEPHADAIYTNP
Haloquadratum    302 --QYDPDYWDSRTYAP------SAFLLYLGVEGDVGDLAHHSLVLPTDWDDHFEQIFDDP
Natrinema        295 --GHDADYWDDRTYAP------SAFLLYLGVEGDVDDLAHHTLVLPTDWQDHFDQIFEDP

KH105_crtIBY1    403 TWAEEPVFYVSATSKTDPSVVTGQGEALFVLVPISYQLNG--TDNAARREQILHTVLTRM
KH105_crtIBY2    403 TWAEEPVFYVSATSKTDPSVVSGQGEALFVLVPISYQLNG--TDNAARREHILHTVLTRM
Brevundimonas    341 DLPQDDFSLYLHAPTRTDPSLAPEGCDAFYVLAPVPHLASAD-IDWAVEGPRYRDRVLAYL
Xanthophyllomyc  370 GLPADPSFYVNVPSRIDPSAAPEGKDAIVILVPCGHIDASNPQDYNKLVARARKFVIHTL
Arabidopsis      428 KEYYDPNRSMLELVFAPAEEWISRTDSDIIDATMKELEKL--FPDEISADQSKAKILKYH
Haematococcus    406 KEYADEKKSMLELVFAPAKEWIGRPDEEIIAATMTELERL--FPTEVRADQSMAKILKYK
Thalassiosira    418 VTAEENCALVSIPSVHDNTLAPDNHAVLHIYTPATELYERWENVKRNTPEYNQLKEERSA
Ectocarpus       436 KGYRDDDKSMLELVFAPAKDWIGKSDEDIIEATMGELYRL--FPNELSKDGSGAKLLKSA
Chlamydomonas    407 KEYYDTEKSMLELVFAPAKDWIGRSDEDIIAATMTELERL--FPTEIKADQSLAKIRKYK
Synechococcus    339 KEYEDPDKSMLELVFAPAKDWIGRPDEEIIEATMSELHKL--FPMHFGGDNP-ATLLKYK
Haliscomenobact  344 RWPEKPLFYVCAPSITDPTVAPEGHENLFVLIPVAPNLE----DTEARREQYYNLVMERL
Saprospira       344 KWPSKPLFYACLASKTDPSCAPEGHENLFLLVPVAPELE----DTEATREHYYNLVMDRL
Fulvivirga       344 GWPQKPLFYVCCTSKTDPGCAPEGMENLFVLIPVAPGLE----DRMEIRERYYDLVMDRI
Pedobacter       353 QWPSKPLFYVCCPSKTDPDVAPEGHENIFILMPLAVGLE----DNDTLRNTYFNSIMTRL
Cyclobacterium   346 RWPEKPLFYASVPSITDATVAPEGMENLFLLIPLAPDLE----DSEATRNKYFNIIMSRL
Haloquadratum    354 AWPADPAYYLCVPSQTDDDVAPDGHSNLFALVPIAAGLD----DSEEHRQRYRNLILDDI
Natrinema        347 QWPDDPAYYLCVPSRTDDDVAPEGHSALFVLVPIAPGLE----DTPAIRDEYRDVILDDI
```

```
KH105_crtIBY1    461 EENLKQ-PLREWLVVYQKSYGTTDFERDFHSFRGNAFGHANTLSQSLVLKPSMDS-LLNNL
KH105_crtIBY2    461 EENLKQ-PLREWLVVYQKSYGTTDFERDFHSFRGNAFGHANTLSQSLVLKPSMDS-LLNNL
Brevundimonas    400 EQHYIP-GLTAHLDTCRIFTPVDFRDQLNAHQGSAFSLEPILTQSAYFRVHNRDDQIPNL
Xanthophyllomyc  430 SAKLGLPDFEKMIVAEKVHDAPSWEKEFNLKDGSILGLAHNFMQVLGFRPSTRHPKYDKL
Arabidopsis      486 VVKTPR-SVYKTIPNCEPCRPLQRSPIEGFYLAGDYTKQKYLAS---MEGAVLSGKFCSQ
Haematococcus    464 VVKTPL-SVYKSTAGREKFRPTQRSPISNFYLAGDYTKQKYLAS---MEGAVFSGKLVTE
Thalassiosira    478 FLWKVLEKIIPDIRQRAVHSKVGTPLTHQRFLNRYRGSYGPAIRAGDASFPFPNTPIQGL
Ectocarpus       494 VVKTPL-SVYEATAGRELYRPVQTTPVSNFFLAGCFTKQKYLAS---MEGATFSGKLAAR
Chlamydomonas    465 VIKTPL-SVYESRAGREAFRPSQRTPIKNFFLAGDFTKQKYLAS---MEGAIFSGKLAAE
Synechococcus    396 VVKTPL-SVYKTTPGCQQLRPDQTTPIKNFFLAGDYTMQRYLAS---MEGAVLSGKLCAG
Haliscomenobact  400 ERLTGQ-EIRSAVVYKRSYAHADFETDYHAFKGNAYGLANTLFQTAFLKPKLRSKKIKNL
Saprospira       400 EALTGQ-EIRSAVDYKLSYAHKDFKKDYYAYKGNAYGLANTLSQTAFLKPRLKNKKLNNF
Fulvivirga       400 EKLTGE-ALKEHIVYKRSYAHNDFIEDYNAFRGNAYGLANTLRQTAILKPSIHNRKVRNL
Pedobacter       409 ETFTGM-AIREHIDYQKSYCVSDFVSDYNSYKGNAYGLANTLMQTANLKPSLKNKKIDNL
Cyclobacterium   402 EKHTGQ-DIRSHIVYNRSYAHSDFKKDYHAFKGNAYGLANTLRQTAILKPSLRNKKLTNL
Haloquadratum    410 AEHTGV-DLRDRIVVERSFCVDDFADRYNSTAGTALGLAHTLEQTALLRPGHHSSAVDGL
Natrinema        403 AENTGT-DLRDRIVLEERFSVADFAGRYNSYGGTALGLAHTLRQTALFRPPHRSAEVDGL

KH105_crtIBY1    519 VFAGHLTNPGPGVPPSIVSGTVSANLLHDKLQVTANHHLLGFTLLGALLGALFLGILTLS
KH105_crtIBY2    519 VFAGHLTNPGPGVPPSIVSGTVSANLLHDKLQVTANHHSLGFALLGALLGALFLGILALS
Brevundimonas    459 YFVGAGTHPGAGVPGVVGSAKATAGLMIEDAGRTA-------------------------
Xanthophyllomyc  490 FFVGASTHPGTGVPIVLAGAKLTANQVLESFDRSPAPDPNMSLSVPYGKPLKSNGTGIDS
Arabidopsis      542 SIVQDYELLAASGPRKLSEATVSSS-----------------------------------
Haematococcus    520 AIVEDWSARGVTSSAASRQPALAAAGVVAGSAAVIGAALVAASGALAGGM----------
Thalassiosira    538 LLCGDSCFPGIGVPAVAGSGMIAANSVSLDSIGAQLEVLSKIKQQ---------------
Ectocarpus       550 ALCD--AAIAGTVPLQKESSAVAA------------------------------------
Chlamydomonas    521 QIVNDYNYKGVAPPARSSSSPELVAASALLAVAAVGAGLVGFGR----------------
Synechococcus    452 AVDQKRDQLSSSTP--VSEPVAA-------------------------------------
Haliscomenobact  459 FFTGQLTTPGPGVPPSLISGQVVAREVSKLLQ----------------------------
Saprospira       459 YYAGQLTTPGPGVPPSLISGEVVAREIIKSKG----------------------------
Fulvivirga       459 FYTGQLTVPGPGVPPSIISGQVVAGEVIGRYEI---------------------------
Pedobacter       468 FYAGQLTVPGPGVPPSIVSGNVAAQQLIKYLNR---------------------------
Cyclobacterium   461 YYSGQLTVPGPGVPPSLISGEVVAKEIIKNTINK--------------------------
Haloquadratum    469 YFTGSFTTPGIGVPMCLISGQLTAEKMADRAT----------------------------
Natrinema        462 YFVGGDTTPGIGVPMCLISGDLTAEKVLEDHGLEG-------------------------

KH105_crtIBY1    579 VFSTRFVSYVECIRLLYVHGRTYFAAATLMKPMAFLDTAAMYGLFRVADDYVDNVGDAGE
KH105_crtIBY2    579 VFSTRFVSYVECIRLLYVHGRTYFAAATLMKPMAFLDTAAMYGLFRVADDYVDNVGDAGE
Brevundimonas        ------------------------------------------------------------
Xanthophyllomyc  550 QVQLKFMDLERWVYLLVLLIGAVIARSVGVLAF---------------------------
Arabidopsis          ------------------------------------------------------------
Haematococcus        ------------------------------------------------------------
Thalassiosira        ------------------------------------------------------------
Ectocarpus           ------------------------------------------------------------
Chlamydomonas        ------------------------------------------------------------
Synechococcus        ------------------------------------------------------------
Haliscomenobact      ------------------------------------------------------------
Saprospira           ------------------------------------------------------------
Fulvivirga           ------------------------------------------------------------
Pedobacter           ------------------------------------------------------------
Cyclobacterium       ------------------------------------------------------------
Haloquadratum        ------------------------------------------------------------
Natrinema            ------------------------------------------------------------
```

```
KH105_crtIBY1     639 RQRNLDAFMADFWRCWESGRGDYARHPTLPAIIESAHRRAYPRELFERFFRSMRMDAKRK
KH105_crtIBY2     639 RQRNLDAFMADFWRCWESGRGDYARHPTLPAIIESAHRRAYPRELFERFFRSMRMDAKRK
Brevundimonas         ------------------------------------------------------------
Xanthophyllomyc       ------------------------------------------------------------
Arabidopsis           ------------------------------------------------------------
Haematococcus         ------------------------------------------------------------
Thalassiosira         ------------------------------------------------------------
Ectocarpus            ------------------------------------------------------------
Chlamydomonas         ------------------------------------------------------------
Synechococcus         ------------------------------------------------------------
Haliscomenobact       ------------------------------------------------------------
Saprospira            ------------------------------------------------------------
Fulvivirga            ------------------------------------------------------------
Pedobacter            ------------------------------------------------------------
Cyclobacterium        ------------------------------------------------------------
Haloquadratum         ------------------------------------------------------------
Natrinema             ------------------------------------------------------------

KH105_crtIBY1     699 VVCLTMDDTMEYMEGSAAVIGEFMLPILMPDRDSLAFKQAIPHARDLGLAFQITNMLRDI
KH105_crtIBY2     699 VVCLTMDDTMEYMEGSAAVIGEFMLPILMPDRDSLAFKQAIPHARDLGLAFQITNMLRDI
Brevundimonas         ------------------------------------------------------------
Xanthophyllomyc       ------------------------------------------------------------
Arabidopsis           ------------------------------------------------------------
Haematococcus         ------------------------------------------------------------
Thalassiosira         ------------------------------------------------------------
Ectocarpus            ------------------------------------------------------------
Chlamydomonas         ------------------------------------------------------------
Synechococcus         ------------------------------------------------------------
Haliscomenobact       ------------------------------------------------------------
Saprospira            ------------------------------------------------------------
Fulvivirga            ------------------------------------------------------------
Pedobacter            ------------------------------------------------------------
Cyclobacterium        ------------------------------------------------------------
Haloquadratum         ------------------------------------------------------------
Natrinema             ------------------------------------------------------------

KH105_crtIBY1     759 GEDNRLGRQYIPVDACNRHGLNGKLTSHEQPGFRELMEEMFAFTDNLYASADLGIDMLPE
KH105_crtIBY2     759 GEDNRLGRQYIPVDACKRHGLNGKLTSHEQPGFRELMEEMFAFTDNLYASADLGIDMLPE
Brevundimonas         ------------------------------------------------------------
Xanthophyllomyc       ------------------------------------------------------------
Arabidopsis           ------------------------------------------------------------
Haematococcus         ------------------------------------------------------------
Thalassiosira         ------------------------------------------------------------
Ectocarpus            ------------------------------------------------------------
Chlamydomonas         ------------------------------------------------------------
Synechococcus         ------------------------------------------------------------
Haliscomenobact       ------------------------------------------------------------
Saprospira            ------------------------------------------------------------
Fulvivirga            ------------------------------------------------------------
Pedobacter            ------------------------------------------------------------
Cyclobacterium        ------------------------------------------------------------
Haloquadratum         ------------------------------------------------------------
Natrinema             ------------------------------------------------------------
```

```
KH105_crtIBY1     819 QVRDVIRVARLAYHRIHDKIRAANYDIFTARRRVPLREKLTILVDTVPRLKLARIALTEL
KH105_crtIBY2     819 QVRDVIRVARLAYHRIHDKIRAANYDIFTARRRVPLREKLTILVDTVPRLKLARIALTEL
Brevundimonas         ------------------------------------------------------------
Xanthophyllomyc       ------------------------------------------------------------
Arabidopsis           ------------------------------------------------------------
Haematococcus         ------------------------------------------------------------
Thalassiosira         ------------------------------------------------------------
Ectocarpus            ------------------------------------------------------------
Chlamydomonas         ------------------------------------------------------------
Synechococcus         ------------------------------------------------------------
Haliscomenobact       ------------------------------------------------------------
Saprospira            ------------------------------------------------------------
Fulvivirga            ------------------------------------------------------------
Pedobacter            ------------------------------------------------------------
Cyclobacterium        ------------------------------------------------------------
Haloquadratum         ------------------------------------------------------------
Natrinema             ------------------------------------------------------------

KH105_crtIBY1     879 ICAALYGLSRPHIAFVWIGAVWASWLEWPGCSYLRFHGLFVVPPLLMLARLAHQRAVADK
KH105_crtIBY2     879 ICAALYGLSRPHIAFVWIGAVWASWLEWPGCSYLRFHGLFVIPPLLLLARLAHQRAVADK
Brevundimonas         ------------------------------------------------------------
Xanthophyllomyc       ------------------------------------------------------------
Arabidopsis           ------------------------------------------------------------
Haematococcus         ------------------------------------------------------------
Thalassiosira         ------------------------------------------------------------
Ectocarpus            ------------------------------------------------------------
Chlamydomonas         ------------------------------------------------------------
Synechococcus         ------------------------------------------------------------
Haliscomenobact       ------------------------------------------------------------
Saprospira            ------------------------------------------------------------
Fulvivirga            ------------------------------------------------------------
Pedobacter            ------------------------------------------------------------
Cyclobacterium        ------------------------------------------------------------
Haloquadratum         ------------------------------------------------------------
Natrinema             ------------------------------------------------------------

KH105_crtIBY1     939 QVPFLRRAGFWTLALCVVATLYTTPWDNFLVYRRVWGYPPERILFVIGYVPVEEYMFFTL
KH105_crtIBY2     939 QVPFLRRAGFWTLALCVVATLYTTPWDNFLVYRRVWGYPPERILFVIGYVPIEEYMFFTL
Brevundimonas         ------------------------------------------------------------
Xanthophyllomyc       ------------------------------------------------------------
Arabidopsis           ------------------------------------------------------------
Haematococcus         ------------------------------------------------------------
Thalassiosira         ------------------------------------------------------------
Ectocarpus            ------------------------------------------------------------
Chlamydomonas         ------------------------------------------------------------
Synechococcus         ------------------------------------------------------------
Haliscomenobact       ------------------------------------------------------------
Saprospira            ------------------------------------------------------------
Fulvivirga            ------------------------------------------------------------
Pedobacter            ------------------------------------------------------------
Cyclobacterium        ------------------------------------------------------------
Haloquadratum         ------------------------------------------------------------
Natrinema             ------------------------------------------------------------
```

```
KH105_crtIBY1     999 ETMLVAAVWLQVFQPTTLQAEVGPRGKGGMLVLASLGLVWIAGLSCLASEQSLYIGLILT
KH105_crtIBY2     999 ETMLVAAVWLQVFQPTTLQAEVGPRGKGGMLVLASLGLVWMAGLSCLASEQSLYIGLILT
Brevundimonas         ------------------------------------------------------------
Xanthophyllomyc       ------------------------------------------------------------
Arabidopsis           ------------------------------------------------------------
Haematococcus         ------------------------------------------------------------
Thalassiosira         ------------------------------------------------------------
Ectocarpus            ------------------------------------------------------------
Chlamydomonas         ------------------------------------------------------------
Synechococcus         ------------------------------------------------------------
Haliscomenobact       ------------------------------------------------------------
Saprospira            ------------------------------------------------------------
Fulvivirga            ------------------------------------------------------------
Pedobacter            ------------------------------------------------------------
Cyclobacterium        ------------------------------------------------------------
Haloquadratum         ------------------------------------------------------------
Natrinema             ------------------------------------------------------------

KH105_crtIBY1    1059 WAMPVLILQWSLGAHVLTTHAKPVLTTIVSATAYLCVADAWAIRHGIWRINPANLVLPMG
KH105_crtIBY2    1059 WAMPVLILQWSLGAHVLTTHAKPVLKTIVSATAYLCVADAWAIRHGIWRINPANLVLPMG
Brevundimonas         ------------------------------------------------------------
Xanthophyllomyc       ------------------------------------------------------------
Arabidopsis           ------------------------------------------------------------
Haematococcus         ------------------------------------------------------------
Thalassiosira         ------------------------------------------------------------
Ectocarpus            ------------------------------------------------------------
Chlamydomonas         ------------------------------------------------------------
Synechococcus         ------------------------------------------------------------
Haliscomenobact       ------------------------------------------------------------
Saprospira            ------------------------------------------------------------
Fulvivirga            ------------------------------------------------------------
Pedobacter            ------------------------------------------------------------
Cyclobacterium        ------------------------------------------------------------
Haloquadratum         ------------------------------------------------------------
Natrinema             ------------------------------------------------------------

KH105_crtIBY1    1119 KYALPLEEALFFLVTSIMCTWGLTLAMVLWGKPIGLAVGVGTWARPPRPGRTQLITCGAV
KH105_crtIBY2    1119 KHALPLEEALFFLVTSIMCTWGLTLAMVLWGKPIGLAVGMGTWARPPRPGRSQLITCGAV
Brevundimonas         ------------------------------------------------------------
Xanthophyllomyc       ------------------------------------------------------------
Arabidopsis           ------------------------------------------------------------
Haematococcus         ------------------------------------------------------------
Thalassiosira         ------------------------------------------------------------
Ectocarpus            ------------------------------------------------------------
Chlamydomonas         ------------------------------------------------------------
Synechococcus         ------------------------------------------------------------
Haliscomenobact       ------------------------------------------------------------
Saprospira            ------------------------------------------------------------
Fulvivirga            ------------------------------------------------------------
Pedobacter            ------------------------------------------------------------
Cyclobacterium        ------------------------------------------------------------
Haloquadratum         ------------------------------------------------------------
Natrinema             ------------------------------------------------------------
```

```
KH105_crtIBY1    1179 LVLSISHPALFKMVPALVVTIMRFGFWACALMAGVHLPARGRVLFAAAVVAISCAPAALA
KH105_crtIBY2    1179 LVLSISHPALFKMVPALVVTIMRFGFWACALMAGVHLPARGRVLFVAAVVAISCAPAALA
Brevundimonas         ------------------------------------------------------------
Xanthophyllomyc       ------------------------------------------------------------
Arabidopsis           ------------------------------------------------------------
Haematococcus         ------------------------------------------------------------
Thalassiosira         ------------------------------------------------------------
Ectocarpus            ------------------------------------------------------------
Chlamydomonas         ------------------------------------------------------------
Synechococcus         ------------------------------------------------------------
Haliscomenobact       ------------------------------------------------------------
Saprospira            ------------------------------------------------------------
Fulvivirga            ------------------------------------------------------------
Pedobacter            ------------------------------------------------------------
Cyclobacterium        ------------------------------------------------------------
Haloquadratum         ------------------------------------------------------------
Natrinema             ------------------------------------------------------------

KH105_crtIBY1    1239 PLLAGTVLVGTLGGWHTRDRDDTLPLYKNA
KH105_crtIBY2    1239 PLLAGTVLVGTLGGWHTRDREDTLPLYKNA
Brevundimonas         ------------------------------
Xanthophyllomyc       ------------------------------
Arabidopsis           ------------------------------
Haematococcus         ------------------------------
Thalassiosira         ------------------------------
Ectocarpus            ------------------------------
Chlamydomonas         ------------------------------
Synechococcus         ------------------------------
Haliscomenobact       ------------------------------
Saprospira            ------------------------------
Fulvivirga            ------------------------------
Pedobacter            ------------------------------
Cyclobacterium        ------------------------------
Haloquadratum         ------------------------------
Natrinema             ------------------------------
```
