# Supplementary material for: A Possible Trifunctional β-Carotene Synthase Gene Identified in the Draft Genome of Aurantiochytrium sp. Strain KH105
Source: Genes (Basel). 2018 Apr 9;9(4):200. doi: 10.3390/genes9040200 (PMC5924542; doi:10.3390/genes9040200)
Supplement: Supplementary file 1 [file genes-09-00200-s001.zip › Supplement/Fig. S5.pdf]

```
KH105_crtIBY1       1 MARRASRPVAAVVVVLVVVASACCWQAAADVADAQGARGPGRESDGDRAKKRIAVLGAGY
KH105_crtIBY2       1 MARRASRLVAAIVVVLVVVAPACCRQAAADVADAQGARGPGQESDGGRAKKRIAVLGAGY
Brevundimonas       1 ------------------------------------------------------------
Xanthophyllomyc     1 ------------------------------------------------------------
Arabidopsis         1 ------------------------------------------------------------
Haematococcus       1 ------------------------------------------------------------
Thalassiosira       1 ------------------------------------------------------------
Ectocarpus          1 ------------------------------------------------------------
Chlamydomonas       1 ------------------------------------------------------------
Synechococcus       1 ------------------------------------------------------------
Haliscomenobact     1 ------------------------------------------------------------
Pedobacter          1 ------------------------------------------------------------
Fulvivirga          1 ------------------------------------------------------------
Haloquadratum       1 ------------------------------------------------------------
Natrinema           1 ------------------------------------------------------------
Cyclobacterium      1 ------------------------------------------------------------

KH105_crtIBY1      61 AGLSAACELSRLGHEVVVLEKNDYVGGRAHQFEVEADNGQTFKFDAGPSWYWMPEVFDRF
KH105_crtIBY2      61 AGLSAACELSRLGHEVVVLEKNDYVGGRAHQFEVEADNGQTFKFDAGPSWYWMPEVFDRF
Brevundimonas       1 ------------------------------------------------------------
Xanthophyllomyc     1 ------------------------------------------------------------
Arabidopsis         1 ------------------------------------------------------------
Haematococcus       1 ------------------------------------------------------------
Thalassiosira       1 ------------------------------------------------------------
Ectocarpus          1 ------------------------------------------------------------
Chlamydomonas       1 ------------------------------------------------------------
Synechococcus       1 ------------------------------------------------------------
Haliscomenobact     1 ------------------------------------------------------------
Pedobacter          1 ------------------------------------------------------------
Fulvivirga          1 ------------------------------------------------------------
Haloquadratum       1 ------------------------------------------------------------
Natrinema           1 ------------------------------------------------------------
Cyclobacterium      1 ------------------------------------------------------------

KH105_crtIBY1     121 FARYGRTVQEFYQLERLDPAYRIIRNDHNGEGTVDVPGASSEAFMSWARQMNGDARLVDR
KH105_crtIBY2     121 FARYGRTVQEFYQLERLDPAYRIIRNDHNGEGTVDVPGASSEAFMSWARQMNGDARLVDR
Brevundimonas       1 ------------------------------------------------------------
Xanthophyllomyc     1 ------------------------------------------------------------
Arabidopsis         1 ------------------------------------------------------------
Haematococcus       1 ------------------------------------------------------------
Thalassiosira       1 ------------------------------------------------------------
Ectocarpus          1 ------------------------------------------------------------
Chlamydomonas       1 ------------------------------------------------------------
Synechococcus       1 ------------------------------------------------------------
Haliscomenobact     1 ------------------------------------------------------------
Pedobacter          1 ------------------------------------------------------------
Fulvivirga          1 ------------------------------------------------------------
Haloquadratum       1 ------------------------------------------------------------
Natrinema           1 ------------------------------------------------------------
Cyclobacterium      1 ------------------------------------------------------------

KH105_crtIBY1     181 LMDEAKAKYEEGVFKWIWHPMVSWWEMIDLNLVRAALQYDMFNSFVAHLQKYISSDTLLM
KH105_crtIBY2     181 LMDEAKAKYEEGVFKWIWHPMVSWWEMIDLSLVRAALQYDMFNSFVAHLQKYISSDTLLM
Brevundimonas       1 ------------------------------------------------------------
Xanthophyllomyc     1 ------------------------------------------------------------
Arabidopsis         1 ------------------------------------------------------------
Haematococcus       1 ------------------------------------------------------------
Thalassiosira       1 ------------------------------------------------------------
Ectocarpus          1 ------------------------------------------------------------
Chlamydomonas       1 ------------------------------------------------------------
Synechococcus       1 ------------------------------------------------------------
Haliscomenobact     1 ------------------------------------------------------------
Pedobacter          1 ------------------------------------------------------------
Fulvivirga          1 ------------------------------------------------------------
Haloquadratum       1 ------------------------------------------------------------
Natrinema           1 ------------------------------------------------------------
Cyclobacterium      1 ------------------------------------------------------------
```

Supplementary Figure S5: Alignment of CrtB1 and CrtB2 amino acids within CrtKH105_CrtIBY1 and CrtKH105_CrtIBY2 with those of other eukaryotes.

```
KH105_crtIBY1      241 ILKWPVIFLGASPNGAPALYSMMTYGGHALGTFYPTGGLARPVVAIAELAKDLGVDIQLD
KH105_crtIBY2      241 ILKWPVIFLGASPNGAPALYSMMTYGGHALGTYYPTGGLARPVVAIAELAKDLGVDIQLD
Brevundimonas        1 ------------------------------------------------------------
Xanthophyllomyc      1 -------------------------------------------------MTALAYYQIHL
Arabidopsis          1 ------------------------------------------------------------
Haematococcus        1 ------------------------------------------------------------
Thalassiosira        1 ------------------------------------------------------------
Ectocarpus           1 ------------------------------------------------------------
Chlamydomonas        1 ------------------------------------------------------------
Synechococcus        1 ------------------------------------------------------------
Haliscomenobact      1 ------------------------------------------------------------
Pedobacter           1 ------------------------------------------------------------
Fulvivirga           1 ------------------------------------------------------------
Haloquadratum        1 ------------------------------------------------------------
Natrinema            1 ------------------------------------------------------------
Cyclobacterium       1 ------------------------------------------------------------

KH105_crtIBY1      301 AEVTSFRFDETGRGVQAVCTRNDRCEAVDGVVAAADYHHVEQTLLPPELRRYEQGFWDAQ
KH105_crtIBY2      301 AEVTSFRFDETGRGVQAVCTRNDRCEAVDGVVAAADYHHVEQTLLPPDLRRYEQGFWDAQ
Brevundimonas        1 ------------------------------------------------------------
Xanthophyllomyc     12 IYTLPILGLLGLLTSPILTKFDIYKISILVFIAFSATTPWDSWIIRNGAWTYPSAESGQG
Arabidopsis          1 ------------------------------------------------------------
Haematococcus        1 ------------------------------------------------------------
Thalassiosira        1 ------------------------------------------------------------
Ectocarpus           1 ------------------------------------------------------------
Chlamydomonas        1 ------------------------------------------------------------
Synechococcus        1 ------------------------------------------------------------
Haliscomenobact      1 ------------------------------------------------------------
Pedobacter           1 ------------------------------------------------------------
Fulvivirga           1 ------------------------------------------------------------
Haloquadratum        1 ------------------------------------------------------------
Natrinema            1 ------------------------------------------------------------
Cyclobacterium       1 ------------------------------------------------------------

KH105_crtIBY1      361 VMSPSCVLFYLGFDHRIQGLTHHTFFFDRDLDAHLHAAFDTHTWAEEPVFYVSATSKTDP
KH105_crtIBY2      361 VMSPSCVLFYLGFDHRIQGLTHHTFFFDRDLDAHLHAAFDTHTWAEEPVFYVSATSKTDP
Brevundimonas        1 ------------------------------------------------------------
Xanthophyllomyc     72 VFGTFLDVPYEEYAFFVIQTVITGLVYVLATRHLLPSLALPKTRSSALSLALKALIPLPI
Arabidopsis          1 --------------------MSSSVAVLWVATSSLNPDPMNNCGLVRVLESSRLFSPCQN
Haematococcus        1 -----------------------------------MATPLPTKRTHSIVSVPPAMLHQSL
Thalassiosira        1 --------------------MRISSIVAATLITCDVASAWSSSAFTSPLSIKTQHRSQAS
Ectocarpus           1 ------------------------MVVLPIGGGGAGDATAAAAVAAAAMPATPSRRGGRG
Chlamydomonas        1 ------------------------------------------------MNFRTAHSAQTC
Synechococcus        1 ------------------------------------------------------------
Haliscomenobact      1 ------------------------------------------------------------
Pedobacter           1 ------------------------------------------------------------
Fulvivirga           1 ------------------------------------------------------------
Haloquadratum        1 ------------------------------------------------------------
Natrinema            1 ------------------------------------------------------------
Cyclobacterium       1 ------------------------------------------------------------

KH105_crtIBY1      421 SVVTGQGEALFVLVPISYQLNGTDNAARREQILHTVLTRMEENLKQPLREWLVYQKSYGT
KH105_crtIBY2      421 SVVSGQGEALFVLVPISYQLNGTDNAARREHILHTVLTRMEENLKQPLREWLVYQKSYGT
Brevundimonas        1 ------------------------------------------------------------
Xanthophyllomyc    132 IYLFTAHPSPSPDPLVTDHYFYMRALSLLITPPTMLLAALSGEYAFDWKSGRAKSTIAAI
Arabidopsis         41 QRLNKGKKKQIPTWSSSFVRNRSRRIGVVSSSLVASPSGEIALSSEEKVYNVVLK-----
Haematococcus       26 PGRCRPHSSRCPVAISATLVGPDPRWSIASS--QVVPKQPQ-LKGKDVEEAAMWR-----
Thalassiosira       41 TKRRDGSILMSAVSQKESSSSSSSGQGRTTKEISNELALGVTLDGGPVIDFASVKDTTSR
Ectocarpus          37 GRGRRWALLAAATTLAMPPCARGFALAPQADVRPSRATGASGSGGGGGRYFGRLGGGCCS
Chlamydomonas       13 PARGR----RMAVARATLLRPQSNVSSSAPS--SSAPGLPQTLKGRDVEDYAMWR-----
Synechococcus        1 ------------------------------------------------------------
Haliscomenobact      1 ------------------------------------------------------------
Pedobacter           1 ------------------------------------------------------------
Fulvivirga           1 ------------------------------------------------------------
Haloquadratum        1 ------------------------------------------------------------
Natrinema            1 ------------------------------------------------------------
Cyclobacterium       1 ------------------------------------------------------------
```

```
KH105_crtIBY1       481 TDFERDFHSFRGNAFGHANTLSQSLVLKPSMDSLLNNLVFAGHLTNPGPGVPPSIVSGTV
KH105_crtIBY2       481 TDFERDFHSFRGNAFGHANTLSQSLVLKPSMDSLLNNLVFAGHLTNPGPGVPPSIVSGTV
Brevundimonas         1 ------------------------------------------------------------
Xanthophyllomyc     192 MIPTVYLIWVDYVAVGQDSWSINDEKIVGWRLGGVLPIEEAMFFLLTNLMIVLGLSACDH
Arabidopsis          96 ------------------------------------------------------------
Haematococcus        78 ------------------------------------------------------------
Thalassiosira       101 AEIALADSRKKYEASGATISPNPGGRLMGINDEVVAEVGYEIGEFAEEYLDKESGETVDE
Ectocarpus           97 TLSASELNVNNNNNNNNNANGVAAVRRSGRGAGGYVARGGGGASSALCMSAASTGTAAND
Chlamydomonas        62 ------------------------------------------------------------
Synechococcus         1 ------------------------------------------------------------
Haliscomenobact       1 ------------------------------------------------------------
Pedobacter            1 ------------------------------------------------------------
Fulvivirga            1 ------------------------------------------------------------
Haloquadratum         1 ------------------------------------------------------------
Natrinema             1 ------------------------------------------------------------
Cyclobacterium        1 ------------------------------------------------------------

KH105_crtIBY1       541 SANLLHDKLQVTANHHLLGFTLLGALLGALFLGILTLSVFSTRFVSYVECIRLLYVHGRT
KH105_crtIBY2       541 SANLLHDKLQVTANHHSLGFALLGALLGALFLGILALSVFSTRFVSYVECIRLLYVHGRT
Brevundimonas         1 ---------------------------------------MSDAVLDHSRQSMEQGSK--S
Xanthophyllomyc     252 TQALYLLHGRTIYGNKKMPSSFPLITPPVLSLFFSSRPYSSQPKRDLELAVKLLEEKSRS
Arabidopsis          96 QAALVNKQLRSSSYDLDVKKPQDVVLPGSLS-------LLGEAYDRCGEVCAEYAKT---
Haematococcus        78 CIDLHRR--------LPNGGAPQQASRWTPA-------TLEEAYQRCGQVTSEFAKT---
Thalassiosira       161 LVQKVARYLRSKSNTDTFPEMEEEEAPFTNQEKIRFNRLLSRAYEESGIVTSAFAKT---
Ectocarpus          157 ITKAVDNAKRLTEGDLENLELAKGPAELSRR-----RAVLNQAYQECERITGIFAKT---
Chlamydomonas        62 CIEAHE----------GQRMAVPRGFKWSGG-------VLDQAYEKCGQVTSEYAKT---
Synechococcus         1 ------------------------MMPLAAP-------DLDAAFEACRRETAEWAKT---
Haliscomenobact       1 --------------------------------MMS---LFNSTCMECSQLITQRYST--S
Pedobacter            1 --------------------------------MKE---IFDKLSAECSRITTKRYST--S
Fulvivirga            1 --------------------------------MKE---IFDAVSIRCSRLTTNSYST--S
Haloquadratum         1 --------------------------------------MVRHEQIERSKAIQKRTGK--T
Natrinema             1 ---------------------------------------MQQEHIDASKAIQKRTGR--T
Cyclobacterium        1 --------------------------------MMDPKQLFDDTALECSKLITQRYST--S

KH105_crtIBY1       601 YFAAATLMKPMAFLDTAAMYGLFRVADDYVD---NVGDAGERQRNLDAFMADFWRCWESG
KH105_crtIBY2       601 YFAAATLMKPMAFLDTAAMYGLFRVADDYVD---NVGDAGERQRNLDAFMADFWRCWESG
Brevundimonas        20 FAAAARLFPAAIRDDAWMFYAWCRHCDDEIDGQVLGHGAVGIDPVLAGRKLVELRERTAA
Xanthophyllomyc     312 FFVASAGFPSEVRERLVGLYAFCRVTDDLIDSPEVSSNPHATIDMVSDFLTLLFGPPLHP
Arabidopsis         146 FYLGTLLMTPERRKAIWAIYVWCRRTDELVD---GPNAS-----HITPMALDRWEARLED
Haematococcus       120 FYLGTQLMTPIQAKSIWAIYVWCRRTDELVD---GPNAN-----KITPKALDRWEERLEA
Thalassiosira       218 FYLGTQVLPEPSMKAIWAIYVWCRRTDEIVD---APRPAAHDPNAEMLTDLSEWEIRLER
Ectocarpus          209 FYLGTKFMSPVARKAVWAIYVWCRRTDDIVD---GPRAMVRGK-KSMQKDLTDWATRLEN
Chlamydomonas       102 FYLGTQLMTPAQAKAVWAVYVWCRRTDELVD---GPNAS-----KITPKALDRWEERLEA
Synechococcus        27 FYLGTLLLPPAKRRAIWAIYVWCRRTDELMD---SPEAQSRPV-AELAERLDRWEEKTRA
Haliscomenobact      24 FSMGIKVFDKAYRASIYAIYGFVRFADEIVD---TFHGH-DKAELLQRFKADTYRAIEEG
Pedobacter           24 FSLGIHFLNKKLRHPIYAIYGFVRLADEIVD---SFHDY-DKSFLLDKFKRDCFEAIEHG
Fulvivirga           24 FSLGIYCLNKDIRDPIYSIYGFVRFADEIVD---SFHDY-DKAQLLHKFMEDTYQAIAMK
Haloquadratum        21 FHLATRVLPERVRHATYVLYAFFRVADEVVD---ATDAAPPPQQRERLAELRRQALGKES
Natrinema            20 FYLATRFLPERVRHATHVLYAFFRIADEVVD---DADDADPATQRAQLESLRAQAVGEQA
Cyclobacterium       27 FTLGIKTLCKKLHMPIYGIYGYVRYADEIVD---TFHDK-DKASLLENFRQDTYKAIDEE

KH105_crtIBY1       658 RGD--YARHPTLP--------------------------------------AIIESAHRR
KH105_crtIBY2       658 RGD--YARHPTLP--------------------------------------AIIESAHRR
Brevundimonas        80 ALAGEPQTDPVFT--------------------------------------AFQRVAARH
Xanthophyllomyc     372 SQPDKILSSPLLPPSHPSRPTGMYPLPPPPSLSPAELVQFLTERVPVQYHFAFRLLAKLQ
Arabidopsis         198 LFR-GRPFDMLDA--------------------------------------ALADTVARY
Haematococcus       172 TFA-GRPYNVLDA--------------------------------------ALSDTISKF
Thalassiosira       275 LFDRGEVVDVLDL--------------------------------------PLLDCKVKY
Ectocarpus          265 VWE-GKPKDALDL--------------------------------------ALVDVRENY
Chlamydomonas       154 LFD-GKPYDELDA--------------------------------------ALTDTAAKF
Synechococcus        83 LFN-GTVENDLDA--------------------------------------VMVDTLERF
Haliscomenobact      80 -----ISLNPVLH--------------------------------------SFQLVVNQY
Pedobacter           80 -----ISLNPVLN--------------------------------------SFQQIVNAY
Fulvivirga           80 -----ISLNPILN--------------------------------------SFQQTVNKY
Haloquadratum        78 ------TDDPVLS--------------------------------------AFAELCESY
Natrinema            77 ------PDDPVLE--------------------------------------AFGELRERY
Cyclobacterium       83 -----ISMNPVLH--------------------------------------AFQLIVNKY
```

```
KH105_crtIBY1     678 A-YPRELFERFFRSMRMDAKRK----------VVCLTMDDTMEYMEGSAAVIGEFMLPIL
KH105_crtIBY2     678 A-YPRELFERFFRSMRMDAKRK----------VVCLTMDDTMEYMEGSAAVIGEFMLPIL
Brevundimonas     102 A-IPAEEAMDLLQGFEMDVEG-----------RRYDTLEDTLDYAYHVAGVVGVMMARIM
Xanthophyllomyc   432 GLIPRYPLDELLRGYTTDLIFPLSTEAVQARKTPIETTADLLDYGLCVAGSVAELLVYVS
Arabidopsis       219 P-VDIQPFRDMIEGMRMDLK-----------KSRYQNFDDLYLYCYYVAGTVGLMSVPVM
Haematococcus     193 P-MDIQPFKDMIEGMRMDLH-----------KTRYQTFDELYEYCYRVAGTVGLMTMPVM
Thalassiosira     297 PTLPITPFSDMIRGMLMDIPGLG--------QERYDTWDELHLYCYRVAGTVGLMSMPVF
Ectocarpus        286 PSLPIAPFKDMIDGMIMDVPGIG--------KNRYQTFEELELYCYRVAGTVGLMVLPIL
Chlamydomonas     175 P-LHIQPFRDMIEGMRMDLV-----------KSRYETFDELYEYCYRVAGTVALMCMPIM
Synechococcus     104 P-QDIQPYLDMIEGQRMDLT-----------WTRYPRFDDLKLYCYRVAGTVGLMTQGVM
Haliscomenobact    97 K-IERELIDAFLYSMEMDLH------------HHQYEDHLYQEYIYGSAEVVGLMCLRVW
Pedobacter         97 H-IDRELIELFLKSMEMDLN------------EQEYTPQLYDEYILGSAQVVGLMCLRVF
Fulvivirga         97 E-VDLELIDHFLKSMEMDLK------------KVRYDINSFNTYIYGSAEVVGLMCLKVF
Haloquadratum      94 E-IPNEDVNVFIDAMLADVDT-----------DQYETYDDLETYMDGSAAAVGRMMTAIM
Natrinema          93 G-IDDEEINTFIDAMATDIET-----------SRYETYADLESYMRGSAASVGVMMTAIM
Cyclobacterium    100 H-IERDLIDAFLNSMAMDLD------------FATYNDSKYKEYIYGSAEVVGLMCLRVF

KH105_crtIBY1     727 MPDRDSLAFKQAIP------HARDLGLAFQITNMLRDIGEDNR-LGRQYIPVDACNRHGL
KH105_crtIBY2     727 MPDRDSLAFKQAIP------HARDLGLAFQITNMLRDIGEDNR-LGRQYIPVDACKRHGL
Brevundimonas     150 GVQ----DAPTLRR-------AQDLGLAFQLTNIARDVVEDAK-GGRVYLPGQWLDEAGV
Xanthophyllomyc   492 WASAPSQVPATIEEREAVLVASREMGTALQLVNIARDIKGDAT-EGRFYLPLSFFGLRDE
Arabidopsis       267 GIDPKSKATTES-----VYNAALALGIANQLTNILRDVGEDAR-RGRVYLPQDELAQAGL
Haematococcus     241 GIDPSYKGPMDV-----VYKAALALGTANQLTNILRDVGEDARERNRIYLPMEDLQQFGL
Thalassiosira     349 GCAEGYTDEVAK-------EPALSLGVAFQITNILRDVGEDAEKRERVYLPQRDMERFGV
Ectocarpus        338 GTAEGVSEEDAK-------FPALSLGIALQLTNILRDVGEDAV-RGRIYLPLEDLEKFGV
Chlamydomonas     223 GIEPTYKGQLEP-----VYRAALALGTANQLTNILRDVGEDAYQRNRIYVPLDELDKYGI
Synechococcus     152 GVDGAYTSAPWSDRP-DTSDAAVALGIANQLTNILRDVGEDRG-RGRIYLPLEDLERFGY
Haliscomenobact   144 TNG-DEQQYQNLKA------PARSLGSAFQKINFLRDMKSDFDERGRVYFPGVDFRNFSE
Pedobacter        144 TEG-NEQQYQLLKP------SAMMLGSAFQKVNFLRDLNADYKNLSRTYFPNVDLRAFSN
Fulvivirga        144 CNG-DKTTYEHLKP------YARKLGSAFQKINFLRDLREDYVHMGRSYFPDVDIRVFDE
Haloquadratum     142 DTE----NAETALP------HATALGEAFQMSNFLRDVGEDVKERDRVYLPQETLRQYDV
Natrinema         141 EPD----AEDVAIP------HAVTLGEAFQMTNFLRDVREDIRERDRIYLPQETLRNHGV
Cyclobacterium    147 CEG-DQGNYEHLKH------AACKLGSAFQKVNFLRDLKSDYEDRGRVYFPGVDFNSFDS

KH105_crtIBY1     780 N---------GKLTSHEQPGFRELMEEMFAFTDNLYASADLGIDMLPEQVRDVIRVARLA
KH105_crtIBY2     780 N---------GKLTSHEQPGFRELMEEMFAFTDNLYASADLGIDMLPEQVRDVIRVARLA
Brevundimonas     198 PRD-------QVDQPRHRQAVAHTAQRLVAAAEPYYASARWGLRDLNPRSAWAVATARGV
Xanthophyllomyc   551 SKLAIPTDWTEPRPQDFDKLLSLSPSSTLPSSNASESFRFEWKTYSLPLVAYAEDLAKHS
Arabidopsis       321 SDEDIFA-------GKVTDKWRNFMKMQLKRARMFFDEAEKGVTELSAASRWPVWASLLL
Haematococcus     296 TEQDVLGAVHVPSQGKVSEKWRAFMKFQIARARQCFADAESGVDQLEAKARWPVWSALIL
Thalassiosira     402 TERQIFD-------KVVDENYINLMKFEIARARMYYARALRGVPMLRPESRLPVQLSLDA
Ectocarpus        390 DPENIMN-------GILDSNYKELMRYQIARARFYYKQAARGIPMLSPEGRLPVQASLDM
Chlamydomonas     278 SEKELLTGLHAPTTGAMDDRWRNFMHFQITRARQYFTDAEGGVDLLAPQARWPVWSALIL
Synechococcus     210 SEDDLMA-------GRLNSAWCELMQFQLKRARDWFARSEAGVRWLSRDARWPVWTSLRL
Haliscomenobact   197 E---------DKLKIESD-------------IKADFDHAYTGIVQLPKGVRFGVYLAYVY
Pedobacter        197 V---------EKQLIEVE-------------IEAEFKEALAGIKQLPSSSRSGVYLAYIY
Fulvivirga        197 N---------TKQKIEES-------------IEEDFRIAFEGIRKLPKCARFGVYLSYVY
Haloquadratum     192 ST-------AQILNLEFDENVRAVIQHEQQRTEALYREGVKGIQYLPEDCQLAVLLAAVL
Natrinema         191 DP-------AQIERLEYSESFAGAMAAELKRTEELYREGVAGIRYLPEDCQLPVLLAAVL
Cyclobacterium    200 H---------VKAEIEAD-------------IKQDFDEALIGINQLPKGAMLGVKIAYLY

KH105_crtIBY1     831 YHRIHDKIRAAN-YDIFTARRRVPLREKLTILVDTVPRLKLARIALTELICAALYGLSRP
KH105_crtIBY2     831 YHRIHDKIRAAN-YDIFTARRRVPLREKLTILVDTVPRLKLARIALTELICAALYGLSRP
Brevundimonas     251 YRAIGRHVSRSG-ATAWDGRTSVDKAGKLALVGRGALITLWCKTLDAWREPPPRPALWTH
Xanthophyllomyc   611 YKGIDRLPTEVQAGMRAACASYLLIGREIKVVWKGDVGERRTVAGWRRVRKVLSVVMSGW
Arabidopsis       374 YRRILDEIEAND-YNNFTKRAYVGKVKKIAALPLAYAKSVLKTSSSRLSI----------
Haematococcus     356 YRQILDAIEKND-YDNFSQRAYVSKAKKMASLPLALTRALLPQHRG--------------
Thalassiosira     455 YGKILDKIEENG-YDSLTKRAYVGKWEKLAGIPASWYRTLDIAKAMPLPGDWERPSLEEY
Ectocarpus        443 YGKILTAIEGNN-YDNFRKRAYISKTEKLLTLPFSYAKSLNPGQINTNIDD------SEF
Chlamydomonas     338 YRQILDAIEAND-YDNFSKRAYVPKWRKMVSLPVAYTRALMPARRR--------------
Synechococcus     263 YRGILDAIERVD-YDVFNNRAYVGKVSKLLDLPRSFVLAQAR------------------
Haliscomenobact   235 YTKLFQKIKNSPAHQVQKQRIRVHDGRKVALLLSSAVRNSLNLL----------------
Pedobacter        235 YKELFNKIKRATAEKVMSERIRISNGHKIGLMCDSIIRFKINAL----------------
Fulvivirga        235 YYALFKKIRKTPSGSVLKKRIRIPNQHKFSILFYTYIKHRFNLI----------------
Haloquadratum     245 YADHHRAIERVD-YDVLSRTPSLSTPRKLWLLLKTRMYWTLTPDPVTVFRRVSVVPYPDE
Natrinema         244 YAEHHTVIRNQG-YDVLSREPSLSTARKLWCLAKTRWHWHWNRDPEAVFERVSAVP----
Cyclobacterium    238 YLKLFYKIKSLSPEIITKKRIRIPNARKFALLVSTYFGYKVGWN----------------
```

```
KH105_crtIBY1      890 HIAFVWIGAVWASWLEWPGCSYLRFHGLFVVPPLLMLARLAHQRAVADKQVPFLRRAGFW
KH105_crtIBY2      890 HIAFVWIGAVWASWLEWPGCSYLRFHGLFVIPPLLLLARLAHQRAVADKQVPFLRRAGFW
Brevundimonas      310 I-----------------------------------------------------------
Xanthophyllomyc    671 EGQ---------------------------------------------------------
Arabidopsis            ------------------------------------------------------------
Haematococcus          ------------------------------------------------------------
Thalassiosira      514 EKSLEQMLENKWVEQRGSSATSDYLQSLNK------------------------------
Ectocarpus         496 KLTRFDKLEMERESFPAGEDDDQF------------------------------------
Chlamydomonas          ------------------------------------------------------------
Synechococcus          ------------------------------------------------------------
Haliscomenobact        ------------------------------------------------------------
Pedobacter             ------------------------------------------------------------
Fulvivirga             ------------------------------------------------------------
Haloquadratum      304 YDDVDETDSDARHDREPNRGWSIGTEFVTQRFSNLVRHFTRGE-----------------
Natrinema          299 -----TADHDHRH---PEHGDGVPTRS---------------------------------
Cyclobacterium         ------------------------------------------------------------

KH105_crtIBY1      950 TLALCVVATLYTTPWDNFLVYRRVWGYPPERILFVIGYVPVEEYMFFTLETMLVAAVWLQ
KH105_crtIBY2      950 TLALCVVATLYTTPWDNFLVYRRVWGYPPERILFVIGYVPIEEYMFFTLETMLVAAVWLQ
Brevundimonas          ------------------------------------------------------------
Xanthophyllomyc        ------------------------------------------------------------
Arabidopsis            ------------------------------------------------------------
Haematococcus          ------------------------------------------------------------
Thalassiosira          ------------------------------------------------------------
Ectocarpus             ------------------------------------------------------------
Chlamydomonas          ------------------------------------------------------------
Synechococcus          ------------------------------------------------------------
Haliscomenobact        ------------------------------------------------------------
Pedobacter             ------------------------------------------------------------
Fulvivirga             ------------------------------------------------------------
Haloquadratum          ------------------------------------------------------------
Natrinema              ------------------------------------------------------------
Cyclobacterium         ------------------------------------------------------------

KH105_crtIBY1     1010 VFQPTTLQAEVGPRGKGGMLVLASLGLVWIAGLSCLASEQSLYIGLILTWAMPVLILQWS
KH105_crtIBY2     1010 VFQPTTLQAEVGPRGKGGMLVLASLGLVWMAGLSCLASEQSLYIGLILTWAMPVLILQWS
Brevundimonas          ------------------------------------------------------------
Xanthophyllomyc        ------------------------------------------------------------
Arabidopsis            ------------------------------------------------------------
Haematococcus          ------------------------------------------------------------
Thalassiosira          ------------------------------------------------------------
Ectocarpus             ------------------------------------------------------------
Chlamydomonas          ------------------------------------------------------------
Synechococcus          ------------------------------------------------------------
Haliscomenobact        ------------------------------------------------------------
Pedobacter             ------------------------------------------------------------
Fulvivirga             ------------------------------------------------------------
Haloquadratum          ------------------------------------------------------------
Natrinema              ------------------------------------------------------------
Cyclobacterium         ------------------------------------------------------------

KH105_crtIBY1     1070 LGAHVLTTHAKPVLTTIVSATAYLCVADAWAIRHGIWRINPANLVLPMGKYALPLEEALF
KH105_crtIBY2     1070 LGAHVLTTHAKPVLKTIVSATAYLCVADAWAIRHGIWRINPANLVLPMGKHALPLEEALF
Brevundimonas          ------------------------------------------------------------
Xanthophyllomyc        ------------------------------------------------------------
Arabidopsis            ------------------------------------------------------------
Haematococcus          ------------------------------------------------------------
Thalassiosira          ------------------------------------------------------------
Ectocarpus             ------------------------------------------------------------
Chlamydomonas          ------------------------------------------------------------
Synechococcus          ------------------------------------------------------------
Haliscomenobact        ------------------------------------------------------------
Pedobacter             ------------------------------------------------------------
Fulvivirga             ------------------------------------------------------------
Haloquadratum          ------------------------------------------------------------
Natrinema              ------------------------------------------------------------
Cyclobacterium         ------------------------------------------------------------
```

```
KH105_crtIBY1     1130 FLVTSIMCTWGLTLAMVLWGKPIGLAVGVGTWARPPRPGRTQLITCGAVLVLSISHPALF
KH105_crtIBY2     1130 FLVTSIMCTWGLTLAMVLWGKPIGLAVGMGTWARPPRPGRSQLITCGAVLVLSISHPALF
Brevundimonas          ------------------------------------------------------------
Xanthophyllomyc        ------------------------------------------------------------
Arabidopsis            ------------------------------------------------------------
Haematococcus          ------------------------------------------------------------
Thalassiosira          ------------------------------------------------------------
Ectocarpus             ------------------------------------------------------------
Chlamydomonas          ------------------------------------------------------------
Synechococcus          ------------------------------------------------------------
Haliscomenobact        ------------------------------------------------------------
Pedobacter             ------------------------------------------------------------
Fulvivirga             ------------------------------------------------------------
Haloquadratum          ------------------------------------------------------------
Natrinema              ------------------------------------------------------------
Cyclobacterium         ------------------------------------------------------------

KH105_crtIBY1     1190 KMVPALVVTIMRFGFWACALMAGVHLPARGRVLFAAAVVAISCAPAALAPLLAGTVLVGT
KH105_crtIBY2     1190 KMVPALVVTIMRFGFWACALMAGVHLPARGRVLFVAAVVAISCAPAALAPLLAGTVLVGT
Brevundimonas          ------------------------------------------------------------
Xanthophyllomyc        ------------------------------------------------------------
Arabidopsis            ------------------------------------------------------------
Haematococcus          ------------------------------------------------------------
Thalassiosira          ------------------------------------------------------------
Ectocarpus             ------------------------------------------------------------
Chlamydomonas          ------------------------------------------------------------
Synechococcus          ------------------------------------------------------------
Haliscomenobact        ------------------------------------------------------------
Pedobacter             ------------------------------------------------------------
Fulvivirga             ------------------------------------------------------------
Haloquadratum          ------------------------------------------------------------
Natrinema              ------------------------------------------------------------
Cyclobacterium         ------------------------------------------------------------

KH105_crtIBY1     1250 LGGWHTRDRDDTLPLYKNA
KH105_crtIBY2     1250 LGGWHTRDREDTLPLYKNA
Brevundimonas          -------------------
Xanthophyllomyc        -------------------
Arabidopsis            -------------------
Haematococcus          -------------------
Thalassiosira          -------------------
Ectocarpus             -------------------
Chlamydomonas          -------------------
Synechococcus          -------------------
Haliscomenobact        -------------------
Pedobacter             -------------------
Fulvivirga             -------------------
Haloquadratum          -------------------
Natrinema              -------------------
Cyclobacterium         -------------------
```
