# Supplementary material for: A Possible Trifunctional β-Carotene Synthase Gene Identified in the Draft Genome of Aurantiochytrium sp. Strain KH105
Source: Genes (Basel). 2018 Apr 9;9(4):200. doi: 10.3390/genes9040200 (PMC5924542; doi:10.3390/genes9040200)
Supplement: Supplementary file 1 [file genes-09-00200-s001.zip › Supplement/Fig. S7.pdf]

```
KH105_crtIBY1        1 MARRASRPVAAVVVVLVVVASACCWQAAADVADAQGARGPGRESDGDRAKKRIAVLGAGY
KH105_crtIBY2        1 MARRASRLVAAIVVVLVVVAPACCRQAAADVADAQGARGPGQESDGGRAKKRIAVLGAGY
Brevundimonas        1 ------------------------------------------------------------
Xanthophyllomyc      1 ------------------------------------------------------------
Arabidopsis          1 ------------------------------------------------------------
Haematococcus        1 ------------------------------------------------------------
Thalassiosira        1 ------------------------------------------------------------
Ectocarpus           1 ------------------------------------------------------------
Chlamydomonas        1 ------------------------------------------------------------
Synechococcus        1 ------------------------------------------------------------
Haloquadratum        1 ------------------------------------------------------------
Natrinema            1 ------------------------------------------------------------
Phycomyces           1 ------------------------------------------------------------
Sporisorium          1 ------------------------------------------------------------
Ustilago             1 ------------------------------------------------------------

KH105_crtIBY1       61 AGLSAACELSRLGHEVVVLEKNDYVGGRAHQFEVEADNGQTFKFDAGPSWYWMPEVFDRF
KH105_crtIBY2       61 AGLSAACELSRLGHEVVVLEKNDYVGGRAHQFEVEADNGQTFKFDAGPSWYWMPEVFDRF
Brevundimonas        1 ------------------------------------------------------------
Xanthophyllomyc      1 ------------------------------------------------------------
Arabidopsis          1 ------------------------------------------------------------
Haematococcus        1 ------------------------------------------------------------
Thalassiosira        1 ------------------------------------------------------------
Ectocarpus           1 ------------------------------------------------------------
Chlamydomonas        1 ------------------------------------------------------------
Synechococcus        1 ------------------------------------------------------------
Haloquadratum        1 ------------------------------------------------------------
Natrinema            1 ------------------------------------------------------------
Phycomyces           1 ------------------------------------------------------------
Sporisorium          1 ------------------------------------------------------------
Ustilago             1 ------------------------------------------------------------

KH105_crtIBY1      121 FARYGRTVQEFYQLERLDPAYRIIRNDHNGEGTVDVPGASSEAFMSWARQMNGDARLVDR
KH105_crtIBY2      121 FARYGRTVQEFYQLERLDPAYRIIRNDHNGEGTVDVPGASSEAFMSWARQMNGDARLVDR
Brevundimonas        1 ------------------------------------------------------------
Xanthophyllomyc      1 ------------------------------------------------------------
Arabidopsis          1 ------------------------------------------------------------
Haematococcus        1 ------------------------------------------------------------
Thalassiosira        1 ------------------------------------------------------------
Ectocarpus           1 ------------------------------------------------------------
Chlamydomonas        1 ------------------------------------------------------------
Synechococcus        1 ------------------------------------------------------------
Haloquadratum        1 ------------------------------------------------------------
Natrinema            1 ------------------------------------------------------------
Phycomyces           1 ------------------------------------------------------------
Sporisorium          1 ------------------------------------------------------------
Ustilago             1 ------------------------------------------------------------

KH105_crtIBY1      181 LMDEAKAKYEEGVFKWIWHPMVSWWEMIDLNLVRAALQYDMFNSFVAHLQKYISSDTLLM
KH105_crtIBY2      181 LMDEAKAKYEEGVFKWIWHPMVSWWEMIDLSLVRAALQYDMFNSFVAHLQKYISSDTLLM
Brevundimonas        1 ------------------------------------------------------------
Xanthophyllomyc      1 ------------------------------------------------------------
Arabidopsis          1 ------------------------------------------------------------
Haematococcus        1 ------------------------------------------------------------
Thalassiosira        1 ------------------------------------------------------------
Ectocarpus           1 ------------------------------------------------------------
Chlamydomonas        1 ------------------------------------------------------------
Synechococcus        1 ------------------------------------------------------------
Haloquadratum        1 ------------------------------------------------------------
Natrinema            1 ------------------------------------------------------------
Phycomyces           1 ------------------------------------------------------------
Sporisorium          1 ------------------------------------------------------------
Ustilago             1 ------------------------------------------------------------
```

Supplementary Figure S7: Alignment of CrtY1 and CrtIY2 amino acids within CrtKH105_CrtIBY1 and CrtKH105_CrtIBY2 with those of other eukaryotes.

```
KH105_crtIBY1     241 ILKWPVIFLGASPNGAPALYSMMTYGGHALGTFYPTGGLARPVVAIAELAKDLGVDIQLD
KH105_crtIBY2     241 ILKWPVIFLGASPNGAPALYSMMTYGGHALGTYYPTGGLARPVVAIAELAKDLGVDIQLD
Brevundimonas       1 ------------------------------------------------------------
Xanthophyllomyc     1 ------------------------------------------------------------
Arabidopsis         1 ------------------------------------------------------------
Haematococcus       1 ------------------------------------------------------------
Thalassiosira       1 ------------------------------------------------------------
Ectocarpus          1 ------------------------------------------------------------
Chlamydomonas       1 ------------------------------------------------------------
Synechococcus       1 ------------------------------------------------------------
Haloquadratum       1 ------------------------------------------------------------
Natrinema           1 ------------------------------------------------------------
Phycomyces          1 ------------------------------------------------------------
Sporisorium         1 ------------------------------------------------------------
Ustilago            1 ------------------------------------------------------------

KH105_crtIBY1     301 AEVTSFRFDETGRGVQAVCTRNDRCEAVDGVVAAADYHHVEQTLLPPELRRYEQGFWDAQ
KH105_crtIBY2     301 AEVTSFRFDETGRGVQAVCTRNDRCEAVDGVVAAADYHHVEQTLLPPDLRRYEQGFWDAQ
Brevundimonas       1 ------------------------------------------------------------
Xanthophyllomyc     1 ------------------------------------------------------------
Arabidopsis         1 ------------------------------------------------------------
Haematococcus       1 ------------------------------------------------------------
Thalassiosira       1 ------------------------------------------------------------
Ectocarpus          1 ------------------------------------------------------------
Chlamydomonas       1 ------------------------------------------------------------
Synechococcus       1 ------------------------------------------------------------
Haloquadratum       1 ------------------------------------------------------------
Natrinema           1 ------------------------------------------------------------
Phycomyces          1 ------------------------------------------------------------
Sporisorium         1 ------------------------------------------------------------
Ustilago            1 ------------------------------------------------------------

KH105_crtIBY1     361 VMSPSCVLFYLGFDHRIQGLTHHTFFFDRDLDAHLHAAFDTHTWAEEPVFYVSATSKTDP
KH105_crtIBY2     361 VMSPSCVLFYLGFDHRIQGLTHHTFFFDRDLDAHLHAAFDTHTWAEEPVFYVSATSKTDP
Brevundimonas       1 ------------------------------------------------------------
Xanthophyllomyc     1 ------------------------------------------------------------
Arabidopsis         1 ------------------------------------------------------------
Haematococcus       1 ------------------------------------------------------------
Thalassiosira       1 ------------------------------------------------------------
Ectocarpus          1 ------------------------------------------------------------
Chlamydomonas       1 ------------------------------------------------------------
Synechococcus       1 ------------------------------------------------------------
Haloquadratum       1 ------------------------------------------------------------
Natrinema           1 ------------------------------------------------------------
Phycomyces          1 ------------------------------------------------------------
Sporisorium         1 ------------------------------------------------------------
Ustilago            1 ------------------------------------------------------------

KH105_crtIBY1     421 SVVTGQGEALFVLVPISYQLNGTDNAARREQILHTVLTRMEENLKQPLREWLVYQKSYGT
KH105_crtIBY2     421 SVVSGQGEALFVLVPISYQLNGTDNAARREHILHTVLTRMEENLKQPLREWLVYQKSYGT
Brevundimonas       1 ------------------------------------------------------------
Xanthophyllomyc     1 ------------------------------------------------------------
Arabidopsis         1 ------------------------------------------------------------
Haematococcus       1 ------------------------------------------------------------
Thalassiosira       1 ------------------------------------------------------------
Ectocarpus          1 ------------------------------------------------------------
Chlamydomonas       1 ------------------------------------------------------------
Synechococcus       1 ------------------------------------------------------------
Haloquadratum       1 ------------------------------------------------------------
Natrinema           1 ------------------------------------------------------------
Phycomyces          1 ------------------------------------------------------------
Sporisorium         1 ------------------------------------------------------------
Ustilago            1 ------------------------------------------------------------
```

```
KH105_crtIBY1      481 TDFERDFHSFRGNAFGHANTLSQSLVLKPSMDSLLNNLVFAGHLTNPGPGVPPSIVSGTV
KH105_crtIBY2      481 TDFERDFHSFRGNAFGHANTLSQSLVLKPSMDSLLNNLVFAGHLTNPGPGVPPSIVSGTV
Brevundimonas        1 ------------------------------------------------------------
Xanthophyllomyc      1 ------------------------------------------------------------
Arabidopsis          1 ------------------------------------------------------------
Haematococcus        1 ------------------------------------------------------------
Thalassiosira        1 ------------------------------------------------------------
Ectocarpus           1 ------------------------------------------------------------
Chlamydomonas        1 ------------------------------------------------------------
Synechococcus        1 ------------------------------------------------------------
Haloquadratum        1 ------------------------------------------------------------
Natrinema            1 ------------------------------------------------------------
Phycomyces           1 ------------------------------------------------------------
Sporisorium          1 ------------------------------------------------------------
Ustilago             1 ------------------------------------------------------------

KH105_crtIBY1      541 SANLLHDKLQVTANHHLLGFTLLGALLGALFLGILTLSVFSTRFVSYVECIRLLYVHGRT
KH105_crtIBY2      541 SANLLHDKLQVTANHHSLGFALLGALLGALFLGILALSVFSTRFVSYVECIRLLYVHGRT
Brevundimonas        1 ------------------------------------------------------------
Xanthophyllomyc      1 ------------------------------------------------------------
Arabidopsis          1 ------------------------------------------------------------
Haematococcus        1 ------------------------------------------------------------
Thalassiosira        1 ------------------------------------------------------------
Ectocarpus           1 ------------------------------------------------------------
Chlamydomonas        1 ------------------------------------------------------------
Synechococcus        1 ------------------------------------------------------------
Haloquadratum        1 ------------------------------------------------------------
Natrinema            1 ------------------------------------------------------------
Phycomyces           1 ------------------------------------------------------------
Sporisorium          1 ------------------------------------------------------------
Ustilago             1 ------------------------------------------------------------

KH105_crtIBY1      601 YFAAATLMKPMAFLDTAAMYGLFRVADDYVDNVGDAGERQRNLDAFMADFWRCWESGRGD
KH105_crtIBY2      601 YFAAATLMKPMAFLDTAAMYGLFRVADDYVDNVGDAGERQRNLDAFMADFWRCWESGRGD
Brevundimonas        1 ------------------------------------------------------------
Xanthophyllomyc      1 ------------------------------------------------------------
Arabidopsis          1 ------------------------------------------------------------
Haematococcus        1 ------------------------------------------------------------
Thalassiosira        1 ------------------------------------------------------------
Ectocarpus           1 ------------------------------------------------------------
Chlamydomonas        1 ------------------------------------------------------------
Synechococcus        1 ------------------------------------------------------------
Haloquadratum        1 ------------------------------------------------------------
Natrinema            1 ------------------------------------------------------------
Phycomyces           1 ------------------------------------------------------------
Sporisorium          1 ------------------------------------------------------------
Ustilago             1 ------------------------------------------------------------

KH105_crtIBY1      661 YARHPTLPAIIESAHRRAYPRELFERFFRSMRMDAKRKVVCLTMDDTMEYMEGSAAVIGE
KH105_crtIBY2      661 YARHPTLPAIIESAHRRAYPRELFERFFRSMRMDAKRKVVCLTMDDTMEYMEGSAAVIGE
Brevundimonas        1 ------------------------------------------------------------
Xanthophyllomyc      1 ------------------------------------------------------------
Arabidopsis          1 ------------------------------------------------------------
Haematococcus        1 ------------------------------------------------------------
Thalassiosira        1 ------------------------------------------------------------
Ectocarpus           1 ------------------------------------------------------------
Chlamydomonas        1 ------------------------------------------------------------
Synechococcus        1 ------------------------------------------------------------
Haloquadratum        1 ------------------------------------------------------------
Natrinema            1 ------------------------------------------------------------
Phycomyces           1 ------------------------------------------------------------
Sporisorium          1 ------------------------------------------------------------
Ustilago             1 ------------------------------------------------------------
```

```
KH105_crtIBY1      721 FMLPILMPDRDSLAFKQAIPHARDLGLAFQITNMLRDIGEDNRLGRQYIPVDACNRHGLN
KH105_crtIBY2      721 FMLPILMPDRDSLAFKQAIPHARDLGLAFQITNMLRDIGEDNRLGRQYIPVDACKRHGLN
Brevundimonas        1 ------------------------------------------------------------
Xanthophyllomyc      1 ------------------------------------------------------------
Arabidopsis          1 ------------------------------------------------------------
Haematococcus        1 --------------------------------------RTFLSQTAIMLSPLQRLCAVPG
Thalassiosira        1 -------------------------------------------------------MVAVN
Ectocarpus           1 ------------------------------------------------------------
Chlamydomonas        1 ----------------------------------------------MMLKAGNRPVALRS
Synechococcus        1 ------------------------------------------------------------
Haloquadratum        1 ------------------------------------------------------------
Natrinema            1 ------------------------------------------------------------
Phycomyces           1 ------------------------------------------------------------
Sporisorium          1 ------------------------------------------------------------
Ustilago             1 ------------------------------------------------------------

KH105_crtIBY1      781 GKLTSHEQPGFRELMEEMFAFTDNLYASADLGIDMLPEQVRDVIRVARLAYHRIHDKIRA
KH105_crtIBY2      781 GKLTSHEQPGFRELMEEMFAFTDNLYASADLGIDMLPEQVRDVIRVARLAYHRIHDKIRA
Brevundimonas        1 ------------------------------------------------------------
Xanthophyllomyc      1 ------------------------------------------------------------
Arabidopsis          1 ------------------------------------------------------------
Haematococcus       23 SSYSAGTALVLLPPAPRVCLSRQHEQTTQWPRRRLVRASAATASEQRPARSSPSGYIMRD
Thalassiosira        6 PLHAVALAVLPSAAYAFVSPTPVVLSPSSARVTPLSGAPYCTSYRVRHSHSFPTTSIQSR
Ectocarpus           1 ------------MAFDMGEMMEQFMNSVSGGGKTGGGGGENGKRVVY-------------
Chlamydomonas       15 GRSAT------VSPISRVVSRPQQLQRRICT--AAAGQKDAFPSGPYPIPPGPVGHFYRE
Synechococcus        1 ------------------------------------------------------------
Haloquadratum        1 ------------------------------------------------------------
Natrinema            1 ------------------------------------------------------------
Phycomyces           1 ------------------------------------------------------------
Sporisorium          1 ------------------------------------------------------------
Ustilago             1 ------------------------------------------------------------

KH105_crtIBY1      841 ANYDIFTARRRVPLREKLTILVDTVPRLKLARIALTELICAALYGLSRPHIAFVWIGAVW
KH105_crtIBY2      841 ANYDIFTARRRVPLREKLTILVDTVPRLKLARIALTELICAALYGLSRPHIAFVWIGAVW
Brevundimonas        1 ------------MQAAAPDSSAPDLLLLGGGLANGLLALRLSQVRPELDVRIVEAADRLG
Xanthophyllomyc      1 ------------------------------------------------------------
Arabidopsis          1 ------------------------------------------------------------
Haematococcus       83 PMPWPTASDIALTQHDLQATPEVDLIVAGAGPSGIAVAERVAAAGFS----VCVVDPEPL
Thalassiosira       66 TTSSTIFASASNPTLTPTSQDTCDVLVLGSGPAARSIATLLSSKANDKAYDVLLADSNYD
Ectocarpus          37 -----------------------DAAIVGYGPAGGVMATLLAEKHG---LKVCIIDPNLE
Chlamydomonas       67 TEKWPTSETVRLQPHDLNEVDYVDLVVAGAGPAGVAVASRVAAAGFS----VCVVDPEPL
Synechococcus        1 ------------------MAEPVDVLVLGGGPAALCIASELNQWGVAVG---CIAPDPVD
Haloquadratum        1 ------------------------------------------------------------
Natrinema            1 ---------MPGPVPAGDRSNRPRSSVPIVPNQYGHGAIRLPSVLPFTAVPVAARTPTAH
Phycomyces           1 ------------------------------------------------------------
Sporisorium          1 -----------------------------------------------------MTRLYQP
Ustilago             1 -----------------------------------------------------MTSLDEP

KH105_crtIBY1      901 ASWLEWPGCSYLRFHGLFVVP----------PLLMLARLAHQRAVADKQVPFLRRAGFWT
KH105_crtIBY2      901 ASWLEWPGCSYLRFHGLFVIP----------PLLLLARLAHQRAVADKQVPFLRRAGFWT
Brevundimonas       49 G--IHTWSFFEADLTPAQRAWIAP-------LIACRWPGYSVRFP-------AFERRLVT
Xanthophyllomyc      1 -----MTALAYYQIHLIYTLP----------ILGLLGLLTS--------PILTKFDIYKI
Arabidopsis          1 ----------------MDLL----------DCLDTTWSGAVVYVDEG------VKKDLSR
Haematococcus      139 GIWPNNYGAWVDEFEAMGLG----------EYMEIVWPKASVHLSNKP----EGEKFLSR
Thalassiosira      126 RRWAPNYGVWQDEWQSICKLYESFNQPIGNEVIDRLWMSTDCFFGGSFDIAAEQRMRLDR
Ectocarpus          71 KRWIPNYGVWVEEWEALDK---DLQIGLG-DCLDRTWEVTDSFFGGSHGTPADERCRIDR
Chlamydomonas      123 AHWPNNYGVWLDEFQAMGLE----------DCLHVIWPKAKVWLNSEA----DGEKFLNR
Synechococcus       40 ASWPNTYGIWADELKMVGLE----------HLLEHRWSDTVSFFGAGGSTAQDQSHAHGI
Haloquadratum        1 -----MSGLSYAGFHVLFTLP----------PLAYLFVFGT--------TPTRRVARVGI
Natrinema           52 VDPPMTVQFTYFGIHLAFLLP----------PILILGWLASR----RDRAWWGSRPLSGL
Phycomyces           1 -------MLTYMEVHLYFTLP----------VLALLAFLYK--------PFFTTKDRFKY
Sporisorium          8 SQDWPACTLSYRHFHLLWTLP----------LCAVLFLVAR--------PFLTKLDRAKL
Ustilago             8 SQDWPACTLSYRHFHLLWTLP----------VCGVLLVIAR--------PFLTKLDRAKL
```

```
KH105_crtIBY1     951 LALCVVATLYTTPWDNFLVYRRVWGYPPER----ILFVIGYVPVEEYMFFTLETMLVAAV
KH105_crtIBY2     951 LALCVVATLYTTPWDNFLVYRRVWGYPPER----ILFVIGYVPIEEYMFFTLETMLVAAV
Brevundimonas      93 GYCSVTAERFAEAVTQALAGRIVTGAAVVS------AGPTEAVLADG------HRLTARA
Xanthophyllomyc    38 SILVFIAFSATTPWDSWIIRNGAWTYPSAESGQGVFGTFLDVPYEEYAFFVIQTVITGLV
Arabidopsis        29 PYGRVNRKQLKSKMLQKCITNGVKFHQSKVTN--VVHEEANSTVVCS--DG--VKIQASV
Haematococcus     185 PYGRVDRPRLKSMLLKKCAAHGVTFLEGKVDG--VNHGEGRSAVSLA--DG--RSIRGSL
Thalassiosira     186 PYCRIERDVLRRVLSP---ASECTSVNIYSPSGSMVHDESGTSVLIQSKDGDISQVRAKL
Ectocarpus        127 PYARVSRDKMQANLKSRLSAAGVTKIKGKVDAKTLEHSADGTSVCLD--DG--QQIDCRL
Chlamydomonas     169 PFGRVDRPKLKRILLERCVASGVTFLDAKVSG--VSHGGGCSAVKLA--DG--REIRGSL
Synechococcus      90 DYGLFDRAELQRYWLER--ADGVVWHQDTAERVDATSATTSVSCVSG------TTLQARL
Haloquadratum      38 ILMTVVAVIYTTPWDNYLIQRGVWWYGPGD----IFARIWKAPVGEYLFFILQPILTGLW
Natrinema          98 GIMIVLAIVYTTPFTNRLIPVGVWWYGEGT----VLATVWHTPLEEYLFFVLQPILTALW
Phycomyces         36 IFLCTVAFATASPWDNYIVYHKAWSYCPEC----VTAVIGYVPLEEYMFFIIMTLITVTF
Sporisorium        50 ILLPIIAFVWTTPWDNLIVKNRAWFYHRHC----IWFTIGYVPIEEYFFFVIQSLISTLW
Ustilago           50 ILLPIIAFVWTTPWDNLIVRNRAWSYHRHC----IWFTIGYVPIEEYFYFIIQSLISTLW

KH105_crtIBY1    1007 WLQVFQPTTLQAEVGPRGKG----GMLVLASLGLVWIAGLSCLAS------EQSLYIGLI
KH105_crtIBY2    1007 WLQVFQPTTLQAEVGPRGKG----GMLVLASLGLVWMAGLSCLAS------EQSLYIGLI
Brevundimonas     141 VIDGRGPTAAPDLA--------LGFQKFVGLEVRLTAP----------HGLKEPIVMDAC
Xanthophyllomyc    98 YVLATRHLLPSLALPKTRSSALSLALKALIPLPIIYLFTAHPSPSPDPLVTDHYFYMRAL
Arabidopsis        83 VLDATGFSRCLVQYDKPYN---PGYQVAYGIVAEVDGH---------PFDVDKMVFMDWR
Haematococcus     239 VLDATGHVRKLIKFDQKFD---PGYQGAYGIVAEVESH---------PFDLETMLFMDWR
Thalassiosira     243 VVDCTGHESKIVLKDDRMKSIPPGFQIAYGILAEVDETSIPNNDFCGPYFKEAMTLFDYR
Ectocarpus        183 LVDCSGHYSELVERVGEHN---PGVQIAYGAEVEVKDG-------HAPYDENAMLFMDYR
Chlamydomonas     223 VLDATGHSRRLVQYDKKFD---PGFQGAYGIVAEVESH---------PFALDTMLFMDWR
Synechococcus     142 VIDASGSRTPHIRRPDQGP---VAGQAAYGVVGRFSQP---------PIEAGRFVLMDYR
Haloquadratum      94 VTTLT----PDATYRNGDFDRRPRTLGALVWLGFGIIGTVL-------LTVESGFYLGAI
Natrinema         154 LCQVRPSAERSLAIPHTHR--------LLGVAGGLAIAVVGWALR----GDTATYYLGWL
Phycomyces         92 TSLTMRWTLPSFFIRPETPVFQSVCVRYIPIVGFLTIAAKAWASS---IPDSHPFYGACI
Sporisorium       106 CTLLTRWALPNLYLVPSSPKRRRLAT--PAVVVCMLCFVLGLKAA---VPETHSYYFGMI
Ustilago          106 CTLITRWTLPNLYLTPSTPARRPLAP--FAVATCVVCFVLGLQAS---VPATHTYYFGMI

KH105_crtIBY1    1057 LTWAMPVLI-----LQWSLGAHVLTTH------------AKPVLTTIVSATAYLCVADAW
KH105_crtIBY2    1057 LTWAMPVLI-----LQWSLGAHVLTTH------------AKPVLKTIVSATAYLCVADAW
Brevundimonas     183 VDQSGGYR-------------FLYVLPFDDR------TLLIEDTRYTDGDDLDHDLFRTG
Xanthophyllomyc   158 SLLITPPTM-----LLAALSGEYAFDW-KSGR-------AKSTIAAIMIPTVYLIWVDYV
Arabidopsis       131 DKHLDSY---PELKERNSKIPTFLYAMPFSSN-----RIFLEETSLVARPGLRMEDIQER
Haematococcus     287 DEHTQGN---PAMHAANNALPTFLYAMPFSKT-----RIFLEETSLVARPAVGFDELKER
Thalassiosira     303 TDHFPEGSN---ELAKAEKAPTFMYAMPLNGN-----RIFFEETSLVARPALSFQECKDR
Ectocarpus        233 TDYADKAELSPEEVKGLDDTPTFLYAMPMGAAPNGQRRIFFEETSLVARPPISFDLCKER
Chlamydomonas     271 DDHTQAPGL-EAMRAANTALPTFLYAMPFTKN-----LVFLEETSLVSRPAVDFPELKDR
Synechococcus     190 CDHLSAAQR--------QEPPTFLYAMDLGDG-----VFFVEETSLALAPGFSYDVLKQR
Haloquadratum     143 LAWACPVVA-----LQWAVGGGY-----LTRR-------WREWSLAIAIPTVYLWMIDSI
Natrinema         202 FLWAGPVLA-----LQWGFGSTYLWAI------------RRSLLVAIAVPTLYLWLVDRI
Phycomyces        149 LWYVCPVLA-----LLWIGSGEYMLRR------------WKAVLFSIAVPTIFLCWVDQY
Sporisorium       161 TWWSSLPLA-----LLLWGSVDFVSNMGVRAG-------LAPFALSVLAPTFYLWASDVY
Ustilago          161 SWWSSMPLA-----LLLWGSIDFVRNMGVGAG-------FIPFAISVLGPTLYLWSSDIY

KH105_crtIBY1    1100 AIRHGIWRINPANLVLPMGKYALPLEEA-----LFFLVTSIMCTWGLTLAMVLWGKPIGL
KH105_crtIBY2    1100 AIRHGIWRINPANLVLPMGKHALPLEEA-----LFFLVTSIMCTWGLTLAMVLWGKPIGL
Brevundimonas     224 VRDYAAQRGWVIETVLREEEGVLPVALDGDIAAHLKRLGPTALSGLRAGLFHPTTGYSLP
Xanthophyllomyc   205 AVGQDSWSINDEKIVGWRLGGVLPIEEA-----MFFLLTNLMIVLGLSACDHTQALYLLH
Arabidopsis       183 MAARLKHLGINVKRIEEDERCVIPMGGP-----LPVLPQRVVGIGGTAGMVHPSTGYMVA
Haematococcus     339 LDARLKWLGIKVKAVEEEEYCLIPMGGV-----LPTHPQRVLGIGGTAGMVHPSTGFMMS
Thalassiosira     355 CMTRLEHLGITVTKVEEEEFCYIPMGGP-----LPAKDQRVIGFGGAAAMVHPSTGYHLC
Ectocarpus        293 LYRRLKHHGIEVTKVMDEELCYIPMGGA-----MPSLTQRVVAFGGASGLVHAATGYMHV
Chlamydomonas     325 LQARLQHLGIKVTNVLEEEYCLIPMGGV-----LPKHPQRVLAIGGTAGMVHPSTGFMIS
Synechococcus     237 LQQRLDQRGVAITEVIHEEFCLFPMNLP-----LPDRSQPLLAFGGAASMVHPASGYMVG
Haloquadratum     186 AIALGVWTISDQYSTGINLLG-LPIEEA-----VFFLITNILIVYGLILFEWIIDYWYHH
Natrinema         245 AIELGVWVISDTYTTGHALLG-LPIEEA-----LFFLVTTLFVVQGITMYVWVLDRIGGV
Phycomyces        192 AIARGTWDISRRTSTGIMVLPSLPLEEF-----LFFLLIDTVLVFASCATDRAHAIVHIY
Sporisorium       209 ALRRGTWHINEATSLNVFPIPHLPIEEM-----LFFLVTNLILVSACFTFDRCVAICRQS
Ustilago          209 ALRRGTWHINQATSLNIFPIPDLPIEEM-----LFFLVTNVILVSACFTFDRCVAICRLS
```

```
KH105_crtIBY1     1155 AVGVG-------------------------------------------------------
KH105_crtIBY2     1155 AVGMG-------------------------------------------------------
Brevundimonas      284 DAVRLA------------------------------------------------------
Xanthophyllomyc    260 GRTIYG-----------NKKMPSSFPLITPPVLSLFFSSRPYSSQPKRDLELAVKLLEEK
Arabidopsis        238 RTLAAA------------------------------------------------------
Haematococcus      394 RMLGAV------------------------------------------------------
Thalassiosira      410 RAMMGA------------------------------------------------------
Ectocarpus         348 RMLAAS------------------------------------------------------
Chlamydomonas      380 RMMGAA------------------------------------------------------
Synechococcus      292 SLLRRG------------------------------------------------------
Haloquadratum      240 PDAIG-------------------------------------------------------
Natrinema          299 SAWTS-------------------------------------------------------
Phycomyces         247 ITPMN------------HNKVSTWYMDFFYLCWAFLQTDQALSGETLSDLDATWRILREA
Sporisorium        264 VAENAPPLSPSYLPLGSLDTYTKLWAAFVRSDRPPVATSAASASVEPRDLAASLQVLRAA
Ustilago           264 APDHQPPLSPSYLPLGSLATYTKLWAAFVRSDRSSLPTSCASASVEPQDLSASLQVLRAA

KH105_crtIBY1     1160 ---------------------------------TWARPPRPGRTQLITCGAVLVLSISHP
KH105_crtIBY2     1160 ---------------------------------TWARPPRPGRSQLITCGAVLVLSISHP
Brevundimonas      290 --DHLAERIEAAPDGP-----------------ALAQVIRRHARDVWAQRGFYRLLNRML
Xanthophyllomyc    309 SRSFFVASAGFPSEVRERLVGLYAFCRVTDDLIDSPEVSSNPHAT-IDMVSDFLTLLFGP
Arabidopsis        244 --PIVANAIVRYLGSP------------SSNSLRGDQLSAEVWRDLWPIERRRQREFFCF
Haematococcus      400 --PTIADAIVDQLSAPADKAT-SLAARKPGSEAEAEAMSAAVWRAAWPVERIRQRLFNTF
Thalassiosira      416 --GEVAKVIREELEEK--------------NWNPDRAAAR-AYNAIWSPTTIAQRNFAVF
Ectocarpus         354 --GAVSRAIAAELKKD--------------GPGTSEAAARRAYQALWPSKAKLQRDFHVF
Chlamydomonas      386 --PTVADTIVDQLSRPADKASESGAPLRPSSEAEAESMAAAVWAATWPLERVRQRAFFTF
Synechococcus      298 --PDLAKALAEALANR---------------NLGSAALAQRGWQALWPIELVLRHQLYQF
Haloquadratum      245 -----------------------------------PEVSR--------------------
Natrinema          304 ---------------------------------TAPRVPTSSDDR---------------
Phycomyces         295 SASFYTASSVFSFEARQDLGVLYGFCRATDDLADNNDVSVPDRKKQLELVRGFVRQMFDS
Sporisorium        324 SKSFNAASLLLPWDLRTDLGCLYAFCRVADDLVDDDAQGLEAKSSNLDVIRAIVDAIYAD
Ustilago           324 SKSFNAASLLLPWDLRTDLGCLYAFCRVADDLVDDHAQELQAKAKNLDVVRDIVDAVYAD

KH105_crtIBY1     1187 ------------------------------------------------------------
KH105_crtIBY2     1187 ------------------------------------------------------------
Brevundimonas      331 FR----------------------------------------------------------
Xanthophyllomyc    368 PLHPSQPDKILSSPLLPPSHPSRPTGMYPLPPPPSLSPAELVQFLTERVPVQYHFAFRLL
Arabidopsis        290 G-----------------------------------------------------------
Haematococcus      457 G-----------------------------------------------------------
Thalassiosira      459 G-----------------------------------------------------------
Ectocarpus         398 G-----------------------------------------------------------
Chlamydomonas      444 G-----------------------------------------------------------
Synechococcus      341 G-----------------------------------------------------------
Haloquadratum      250 ------------------------------------------------------------
Natrinema              ------------------------------------------------------------
Phycomyces         355 K----------------HGHPDIDWTQYS-----------------GSIPDSFIAAFRSF
Sporisorium        384 SPEAAPQKQPSAQPVSDRIRTLLAPVALP-----------------DKVKQDTRAAAASI
Ustilago           384 S--IVDRKQHLSTPIGERLRILLASAALS-----------------DKIKQDTRAATSI

KH105_crtIBY1     1187 ------------------------------------------------------------
KH105_crtIBY2     1187 ------------------------------------------------------------
Brevundimonas      333 ------------------------------------------------------------
Xanthophyllomyc    428 AKLQGLIPRYPLDELLRGYTTDLIFPLSTEAVQARKTPIETTADLLDYGLCVAGSVAELL
Arabidopsis        291 ------------------------------------------------------------
Haematococcus      458 ------------------------------------------------------------
Thalassiosira      460 ------------------------------------------------------------
Ectocarpus         399 ------------------------------------------------------------
Chlamydomonas      445 ------------------------------------------------------------
Synechococcus      342 ------------------------------------------------------------
Haloquadratum      250 ------------------------------------------------------------
Natrinema              ------------------------------------------------------------
Phycomyces         382 TRLRDVLEIKAVEELLDGYTFDLEQRE-----------VKNEDDLVYYSACVASSVGEMC
Sporisorium        427 APLTRYIPKRLWYEMLQGYSLDLLFEHPDAD---KRTRLRTMDDLVEYSQCVAGVVGEMC
Ustilago           425 APLTQYIPKRLWYEMLQGYSADLRFQHQDAR---QRTRLQSMDDLVEYSQCVAGVVGEMC
```

```
KH105_crtIBY1     1187 ------------------------------------------------------------
KH105_crtIBY2     1187 ------------------------------------------------------------
Brevundimonas      333 ------------------------------------------------------------
Xanthophyllomyc    488 VYVSWASAPSQVPATIEEREAVLVA------------------------------SREMG
Arabidopsis        291 ---------------------------------------------------------MDI
Haematococcus      458 ---------------------------------------------------------MEL
Thalassiosira      460 ---------------------------------------------------------GEF
Ectocarpus         399 ---------------------------------------------------------GEF
Chlamydomonas      445 ---------------------------------------------------------MDV
Synechococcus      342 ---------------------------------------------------------LGR
Haloquadratum      250 ------------------------------------------------------------
Natrinema              ------------------------------------------------------------
Phycomyces         431 TRVLMGSEPGGNRTMLKWTVER---------------------------------ARDMG
Sporisorium        484 TRVILG--RCGGAVPLELKVDRTIAVPSTK---AAMAGKALDLTRADDVHALLYEARRMG
Ustilago           482 IRVILG--RCGSTVPLDLEVDRKIALRASKTIEQSINVKPLDLNRTADVHTLLYEARRMG

KH105_crtIBY1     1187 ---ALFKMVPALVVTIMRFG----------------------------------------
KH105_crtIBY2     1187 ---ALFKMVPALVVTIMRFG----------------------------------------
Brevundimonas      333 --AARPDQRYRVLERFYRLP----------------------------------------
Xanthophyllomyc    518 TALQLVNIARDIKGDATEGRFYLPLSFFGLRDESKLAIPTDWTEPRPQDFDKLLSLSPSS
Arabidopsis        294 LLKLDLDATRRFFDAFFDLQPHYWHGFLSSRLFLP-------------------------
Haematococcus      461 LLSLDLQQTRDFFSAFFNLSDFHWHGFLSTRLSFS-------------------------
Thalassiosira      463 LMKQNVVGLRGFFDGFFKLPLGLWGGFLAGWPGLPNN-----------------------
Ectocarpus         402 LMAQPASTLRGFFNGFFKLPLPLWAGFLSGYPNLPHN-----------------------
Chlamydomonas      448 LLKLNLPQIREFFRAFFSLSDFHWHGFLSTRLSLP-------------------------
Synechococcus      345 LMGFNEALLRTHFATFFSLPREEWFGFLTNTLPLP-------------------------
Haloquadratum      250 ------------------------------------------EWS---------------
Natrinema              ------------------------------------------------------------
Phycomyces         458 LALQLTNIARDIVTDSKQLG-----RSYVPRDWLTSQ-----ESALLK------------
Sporisorium        539 VSLQLVNIARDIVPDSVELR-----RCYLPTDMFDKQ-DARMQDALLAGHIAVR---SQH
Ustilago           540 VSLQLVNIARDVVPDSVELR-----RCYLPADMFDKEEDDGMYEALLKGRVALPRHASKL

KH105_crtIBY1     1204 ----------------------------FWACALMAGVHLPARGRVLFAAAVVAISCAPA
KH105_crtIBY2     1204 ----------------------------FWACALMAGVHLPARGRVLFVAAVVAISCAPA
Brevundimonas      351 --------------------------QPLIERFYAGETTLADKARILSGKPPVPIGAALT
Xanthophyllomyc    578 TLPSSNASESFRFEWKTYSLPLVAYAEDLAKHSYKGIDRLP-TEVQAGMRAACASYLLIG
Arabidopsis        329 ------------------------ELLVFGLSLFSHASNTSRLEIMTKGTVPLAK--MIN
Haematococcus      496 ------------------------QLLGFGITLFFKSSNNIRIHLLKLGVPGLVR--ILF
Thalassiosira      500 -----------------ENHETWWARLVFGLTFVSKLP--VSVAVDMLGSIATYS---IS
Ectocarpus         439 -----------------ELHQDWQSRFKFGLQFFFKLAPAVQFQLALAGALSGWQYGLIK
Chlamydomonas      483 ------------------------QLIVFGLTLFWKSSNQARASLLQLGIPGLVV--MLS
Synechococcus      380 ------------------------RLMGVMLRLFALSPWELRRGLVLGAAMDQLP-----
Haloquadratum      253 -------------------------------------------------------YLLIG
Natrinema              ------------------------------------------------------------
Phycomyces         496 ---AGKARELGDERLRQIALKMVYTADDLNLMASRAIDYLP-PSSRCGVRAACNVYTAIG
Sporisorium        590 TTTLEEKELVQPRDVRKYALRLLRVSRGLYDQAYPALAQIPNRPARAGLKAACSVYAAIG
Ustilago           595 VHGAEEQGVVRPQDVRKYALRLLEISQRLYDESYPSLRQIPNRPARAGLKAACSVYAAIG

KH105_crtIBY1     1236 ALAPLLAGTVLVGTLGGWHTRDRDDTLPLYKNA---------------------------
KH105_crtIBY2     1236 ALAPLLAGTVLVGTLGGWHTRDREDTLPLYKNA---------------------------
Brevundimonas      385 CLVERGRA----------------------------------------------------
Xanthophyllomyc    637 REIKVVW----KGDVGERRTVAGWRRVRKVLSVVMSGWEGQ-------------------
Arabidopsis        363 NLVQDRD-----------------------------------------------------
Haematococcus      530 MLAPTLRG-YYKHAPTVRQQKAVADAAAAVAARTA-AAAKPADLPPAMIK----------
Thalassiosira      538 EGVPLP--QSVTPLLGLPDGYEYREKKSSIGDVAAKHEARRMIMESTVEEVVPVDFEEKT
Ectocarpus         482 AVTPLAEEEEAKDLLFDAEVDAIRAAAKAKGDAASGASAE----GSPVADGKKKEEKERA
Chlamydomonas      517 GLAPTLGGGYYPDTMSLKERKDAVDAAARSAAAAARAAADVASDAAAFVSANSSGADMAV
Synechococcus      411 TFDQSSG-----------------------------------------------------
Haloquadratum      258 REIKVVW----KGDVGERRTVAGWRRVRKVLSVVMSGWEGQ-------------------
Natrinema              ------------------------------------------------------------
Phycomyces         552 VSLHKANGYPDRAHLTKLERMKVTFRCVYGFRKGHQGVQGDRGKSQAFTVI---------
Sporisorium        650 TRIEAQT--ETDVAEGRRARMSNRDRMLRAVSAVYFGV----------------------
Ustilago           655 KRIQAQT--EDEISSGRRARMSNLDRILRAISAVYFDV----------------------
```

```
KH105_crtIBY1           --------------
KH105_crtIBY2           --------------
Brevundimonas           --------------
Xanthophyllomyc         --------------
Arabidopsis             --------------
Haematococcus           --------------
Thalassiosira     596   AA------------
Ectocarpus        538   LAA-----------
Chlamydomonas     577   VEVVEKAFSTSNTK
Synechococcus           --------------
Haloquadratum           --------------
Natrinema               --------------
Phycomyces              --------------
Sporisorium             --------------
Ustilago                --------------
```
