# Supplementary material for: A Possible Trifunctional β-Carotene Synthase Gene Identified in the Draft Genome of Aurantiochytrium sp. Strain KH105
Source: Genes (Basel). 2018 Apr 9;9(4):200. doi: 10.3390/genes9040200 (PMC5924542; doi:10.3390/genes9040200)
Supplement: Supplementary file 1 [file genes-09-00200-s001.zip › Supplement/Table S1.docx]

Supplementary Table S1: Summary of the Roche 454 and Illumina Miseq and GAIIx data used for assembling *Aurantiochytrium* sp. KH105.

|  |  | Used data | | | Assembly | | | | | |
| --- | --- | --- | --- | --- | --- | --- | --- | --- | --- | --- |
|  |  | Total sequences (Gb) | Number of reads (million) | Average read length (bp) | Contig | | | Scaffold | | |
|  |  |  |  |  | Number | N50 (kb) | Total length (Mb) | Number | N50  (kb) | Total length (Mb) |
| 454 | Shotgun | 2.9 | 4.4 | 661 | 2,038 | 71.2 | 76.5 | 512 | 366.9 | 76.7 |
| Miseq | Shotgun | 1.4 | 5.4 | 249 |  |  |  |  |  |  |
| GAIIx | 3-kb mate pair | 4.6 | 30.9 | 148 |  |  |  |  |  |  |
