# Supplementary material for: A Possible Trifunctional β-Carotene Synthase Gene Identified in the Draft Genome of Aurantiochytrium sp. Strain KH105
Source: Genes (Basel). 2018 Apr 9;9(4):200. doi: 10.3390/genes9040200 (PMC5924542; doi:10.3390/genes9040200)
Supplement: Supplementary file 1 [file genes-09-00200-s001.zip › Supplement/Table S1.pdf]

Supplementary Table S1: Summary of the Roche 454 and Illumina Miseq and GAIIx data used for assembling *Aurantiochytrium* sp. KH105.

		Used data			Assembly					
					Contig			Scaffold		
		Total sequences (Gb)	Number of reads (million)	Average read length (bp)	Number	N50 (kb)	Total length (Mb)	Number	N50 (kb)	Total length (Mb)
454	Shotgun	2.9	4.4	661	2,038	71.2	76.5	512	366.9	76.7
Miseq	Shotgun	1.4	5.4	249						
GAIIx	3-kb mate pair	4.6	30.9	148						
