# Supplementary material for: A Possible Trifunctional β-Carotene Synthase Gene Identified in the Draft Genome of Aurantiochytrium sp. Strain KH105
Source: Genes (Basel). 2018 Apr 9;9(4):200. doi: 10.3390/genes9040200 (PMC5924542; doi:10.3390/genes9040200)
Supplement: Supplementary file 1 [file genes-09-00200-s001.zip › Supplement/Table S2.docx]

Supplementary Table S2: RNA-seq data summary for differential gene expression profile analyses

| Sample | #Sequences | Total Bases (Gb) | #Assembled Transcripts |
| --- | --- | --- | --- |
| A_Ia | 28,157,058 | 8.45 | 82,160 |
| A_Ib | 27,715,229 | 8.31 | 112,852 |
| A_Ic | 19,557,035 | 5.87 | 64,365 |
| A_IIa | 25,171,065 | 7.55 | 114,682 |
| A_IIb | 27,209,859 | 8.16 | 109,189 |
| A_IIc | 26,404,861 | 7.92 | 109,917 |
| A_IIIa | 40,394,441 | 12.12 | 101,052 |
| A_IIIb | 24,777,602 | 7.43 | 100,636 |
| A_IIIc | 33,084,528 | 9.93 | 118,491 |
| B_a | 36,097,955 | 10.83 | 79,447 |
| B_b | 32,924,373 | 9.88 | 74,319 |
| B_c | 31,840,576 | 9.55 | 78,507 |
