# Supplementary material for: A Possible Trifunctional β-Carotene Synthase Gene Identified in the Draft Genome of Aurantiochytrium sp. Strain KH105
Source: Genes (Basel). 2018 Apr 9;9(4):200. doi: 10.3390/genes9040200 (PMC5924542; doi:10.3390/genes9040200)
Supplement: Supplementary file 1 [file genes-09-00200-s001.zip › Supplement/Table S2.pdf]

Supplementary Table S2: RNA-seq data summary for differential gene expression profile analyses

Sample	#Sequences	Total Bases (Gb)	#Assembled Transcripts
A_Ia	28,157,058	8.45	82,160
A_Ib	27,715,229	8.31	112,852
A_Ic	19,557,035	5.87	64,365
A_IIa	25,171,065	7.55	114,682
A_IIb	27,209,859	8.16	109,189
A_IIc	26,404,861	7.92	109,917
A_IIIa	40,394,441	12.12	101,052
A_IIIb	24,777,602	7.43	100,636
A_IIIc	33,084,528	9.93	118,491
B_a	36,097,955	10.83	79,447
B_b	32,924,373	9.88	74,319
B_c	31,840,576	9.55	78,507
