# Supplementary material for: A Possible Trifunctional β-Carotene Synthase Gene Identified in the Draft Genome of Aurantiochytrium sp. Strain KH105
Source: Genes (Basel). 2018 Apr 9;9(4):200. doi: 10.3390/genes9040200 (PMC5924542; doi:10.3390/genes9040200)
Supplement: Supplementary file 1 [file genes-09-00200-s001.zip › Supplement/Table S3.docx]

|  | number of 248 ultra-conserved CEGs present in genome | % 248 ultra-conserved CEGs present | total number of CEGs | average number of orthologs per CEG | % detected CEGs that have more than 1 ortholog |
| --- | --- | --- | --- | --- | --- |
| *Auranthiochytrium* sp. KH105 | 225 | 90.73 | 504 | 2.24 | 95.56 |
| *Phytophthora infestans* | 230 | 92.74 | 287 | 1.25 | 22.61 |
| *Phytophthora ramorum* | 229 | 92.34 | 271 | 1.18 | 15.72 |
| *Phytophthora sojae* | 232 | 93.55 | 282 | 1.22 | 18.10 |
| *Saccharomyces cerevisiae* |  |  |  | 1.10 ± 0.35 | 8.8 |
| *Schizosaccharomyces pombe* |  |  |  | 1.11 ± 0.39 | 8.8 |

**Supplementary Table S3: Statistics of the completeness of genomes based on 248 core eukaryotic genes (CEGs)**
