# Supplementary material for: A Possible Trifunctional β-Carotene Synthase Gene Identified in the Draft Genome of Aurantiochytrium sp. Strain KH105
Source: Genes (Basel). 2018 Apr 9;9(4):200. doi: 10.3390/genes9040200 (PMC5924542; doi:10.3390/genes9040200)
Supplement: Supplementary file 1 [file genes-09-00200-s001.zip › Supplement/Table S3.pdf]

Supplementary Table S3: Statistics of the completeness of genomes based on 248 core eukaryotic genes (CEGs)

	number of 248 ultra-conserved CEGs present in genome	% 248 ultra-conserved CEGs present	total number of CEGs	average number of orthologs per CEG	% detected CEGs that have more than 1 ortholog
Auranthiochytrium sp. KH105	225	90.73	504	2.24	95.56
Phytophthora infestans	230	92.74	287	1.25	22.61
Phytophthora ramorum	229	92.34	271	1.18	15.72
Phytophthora sojae	232	93.55	282	1.22	18.10
Saccharomyces cerevisiae				1.10 ± 0.35	8.8
Schizosaccharomyces pombe				1.11 ± 0.39	8.8
