# Supplementary material for: A Possible Trifunctional β-Carotene Synthase Gene Identified in the Draft Genome of Aurantiochytrium sp. Strain KH105
Source: Genes (Basel). 2018 Apr 9;9(4):200. doi: 10.3390/genes9040200 (PMC5924542; doi:10.3390/genes9040200)
Supplement: Supplementary file 1 [file genes-09-00200-s001.zip › Supplement/Table S4r-annotated.docx]

Table S4. List of genes involved in carotenoid biosynthesis characterized by genome decoding and

transcriptome analysis of *Aurantiochytrium* sp. KH105.

| Gene | EC number | Normalized Gene Expression  (log_10_ (FPKM+1)) | | | |
| --- | --- | --- | --- | --- | --- |
|  |  | 28°C, 27 hrs ^1^ | 28°C, 40 hrs ^2^ | 28°C, 48 hrs ^3^ | 16°C, 48 hrs ^1^ |
| Hexokinase | 2.7.1.1 | 1.818 | 1.684 | 1.351 | 1.774 |
| ADP-dependent glucokinase | 2.7.1.147 | 0.668 | 0.590 | 0.684 | 0.730 |
| Glucose-6-phosphate isomerase | 5.3.1.9 | 2.068 | 2.188 | 1.917 | 2.018 |
| 6-Phosphofructokinase | 2.7.1.11 | 2.010 | 2.018 | 1.631 | 1.840 |
| Fructose bisphosphate aldolase | 4.1.2.13 | 2.434 | 2.447 | 1.886 | 2.213 |
| Triosephosphate isomerase | 5.3.1.1.00001 | 2.828 | 2.714 | 2.555 | 2.640 |
|  | 5.3.1.1.000225 | 2.211 | 2.172 | 1.888 | 2.456 |
| Glyceraldehyde-3-phosphate dehydrogenase | 1.2.1.12 | 2.618 | 2.397 | 2.430 | 2.435 |
| Phosphoglycerate kinase | 2.7.2.3 | 1.981 | 1.962 | 1.495 | 1.812 |
| Phosphoglycerate mutase | 5.4.2.1 | 1.653 | 1.616 | 1.446 | 1.518 |
| Phosphopyruvate hydratase | 4.2.1.11 | 2.225 | 2.140 | 1.930 | 2.104 |
| Pyruvate kinase | 2.7.1.40 | 2.234 | 2.288 | 1.841 | 2.264 |
| Pyruvate dehydrogenase E1α | 1.2.4.1 | 2.197 | 2.185 | 2.057 | 2.318 |
| Pyruvate dehydrogenase E1β | 1.2.4.1 | 1.841 | 1.745 | 1.544 | 1.790 |
| Pyruvate dehydrogenase E2 | 2.3.1.12 | 1.570 | 1.500 | 1.237 | 1.497 |
| Dihydrolipoamide dehydrogenase | 1.8.1.4 | 1.810 | 1.738 | 1.524 | 1.560 |
| Acetyl-CoA acetyltransferase | 2.3.1.9 | 1.683 | 1.113 | 1.231 | 1.362 |
| HMG-CoA synthase | 2.3.3.10 | 2.314 | 1.584 | 0.722 | 1.245 |
| HMG-CoA reductase | 1.1.1.34 | 1.772 | 1.537 | 0.752 | 1.376 |
| Mevalonate kinase | 2.7.1.36 | 2.247 | 1.918 | 1.194 | 1.939 |
| Phosphomevalonate kinase | 2.7.4.2 | 1.066 | 0.764 | 0.609 | 0.641 |
| Diphosphomevalonate decarboxylase | 4.1.1.33 | 1.931 | 1.619 | 0.905 | 1.641 |
| Isopentenyl diphosphate isomerase | 5.3.3.2 | 1.251 | 1.027 | 0.917 | 1.264 |
| Geranyl diphosphate synthase | 2.5.1.1 | 2.053 | 2.051 | 2.093 | 2.002 |
| Farnesyl diphosphate synthase | 2.5.1.10 | 2.123 | 1.806 | 1.097 | 1.809 |
| Squalene synthase^4^ | 2.5.1.21 | 1.097 | 1.010 | 0.384 | 0.769 |
| Geranylgeranyl diphosphate synthase (CrtE) | 2.5.1.29 | 2.134 | 2.420 | 2.484 | 2.074 |
| β-carotene synthase (CrtIBY) |  | 0.960 | 1.035 | 1.181 | 0.937 |
| β-carotene ketolase (CrtO) |  | 0.526 | 0.806 | 1.021 | 0.539 |
| β-carotene 3-hydroxylase (CrtZ) | 1.14.13.129 | 0.241 | 0.000 | 1.037 | 0.000 |

^1^ Mid-log Phase

^2^ Late-log to Stationary Phase

^3^ Starvation Phase

^4^ Squalene synthase is not directly involved in the carotenoid biosynthesis but affect the supply of farnesyl diphosphate.
