# Supplementary material for: A Possible Trifunctional β-Carotene Synthase Gene Identified in the Draft Genome of Aurantiochytrium sp. Strain KH105
Source: Genes (Basel). 2018 Apr 9;9(4):200. doi: 10.3390/genes9040200 (PMC5924542; doi:10.3390/genes9040200)
Supplement: Supplementary file 1 [file genes-09-00200-s001.zip › Supplement/Table S4r-annotated.pdf]

Table S4. List of genes involved in carotenoid biosynthesis characterized by genome decoding and transcriptome analysis of *Aurantiochytrium* sp. KH105.

Gene	EC number	Normalized Gene Expression ($\log_{10}$ (FPKM+1))			
		28°C, 27 hrs [1]	28°C, 40 hrs [2]	28°C, 48 hrs [3]	16°C, 48 hrs [1]
Hexokinase	2.7.1.1	1.818	1.684	1.351	1.774
ADP-dependent glucokinase	2.7.1.147	0.668	0.590	0.684	0.730
Glucose-6-phosphate isomerase	5.3.1.9	2.068	2.188	1.917	2.018
6-Phosphofructokinase	2.7.1.11	2.010	2.018	1.631	1.840
Fructose bisphosphate aldolase	4.1.2.13	2.434	2.447	1.886	2.213
Triosephosphate isomerase	5.3.1.1.00001	2.828	2.714	2.555	2.640
	5.3.1.1.000225	2.211	2.172	1.888	2.456
Glyceraldehyde-3-phosphate dehydrogenase	1.2.1.12	2.618	2.397	2.430	2.435
Phosphoglycerate kinase	2.7.2.3	1.981	1.962	1.495	1.812
Phosphoglycerate mutase	5.4.2.1	1.653	1.616	1.446	1.518
Phosphopyruvate hydratase	4.2.1.11	2.225	2.140	1.930	2.104
Pyruvate kinase	2.7.1.40	2.234	2.288	1.841	2.264
Pyruvate dehydrogenase E1α	1.2.4.1	2.197	2.185	2.057	2.318
Pyruvate dehydrogenase E1β	1.2.4.1	1.841	1.745	1.544	1.790
Pyruvate dehydrogenase E2	2.3.1.12	1.570	1.500	1.237	1.497
Dihydrolipoamide dehydrogenase	1.8.1.4	1.810	1.738	1.524	1.560
Acetyl-CoA acetyltransferase	2.3.1.9	1.683	1.113	1.231	1.362

HMG-CoA synthase	2.3.3.10	2.314	1.584	0.722	1.245
HMG-CoA reductase	1.1.1.34	1.772	1.537	0.752	1.376
Mevalonate kinase	2.7.1.36	2.247	1.918	1.194	1.939
Phosphomevalonate kinase	2.7.4.2	1.066	0.764	0.609	0.641
Diphosphomevalonate decarboxylase	4.1.1.33	1.931	1.619	0.905	1.641
Isopentenyl diphosphate isomerase	5.3.3.2	1.251	1.027	0.917	1.264
Geranyl diphosphate synthase	2.5.1.1	2.053	2.051	2.093	2.002
Farnesyl diphosphate synthase	2.5.1.10	2.123	1.806	1.097	1.809
Squalene synthase[4]	2.5.1.21	1.097	1.010	0.384	0.769
Geranylgeranyl diphosphate synthase (CrtE)	2.5.1.29	2.134	2.420	2.484	2.074
β-carotene synthase (CrtIBY)		0.960	1.035	1.181	0.937
β-carotene ketolase (CrtO)		0.526	0.806	1.021	0.539
β-carotene 3-hydroxylase (CrtZ)	1.14.13.129	0.241	0.000	1.037	0.000

[1] Mid-log Phase

[2] Late-log to Stationary Phase

[3] Starvation Phase

[4] Squalene synthase is not directly involved in the carotenoid biosynthesis but affect the supply of farnesyl diphosphate.
