# Supplementary figures and images for: A Possible Trifunctional β-Carotene Synthase Gene Identified in the Draft Genome of Aurantiochytrium sp. Strain KH105
Source: Genes (Basel). 2018 Apr 9;9(4):200. doi: 10.3390/genes9040200 (PMC5924542; doi:10.3390/genes9040200)

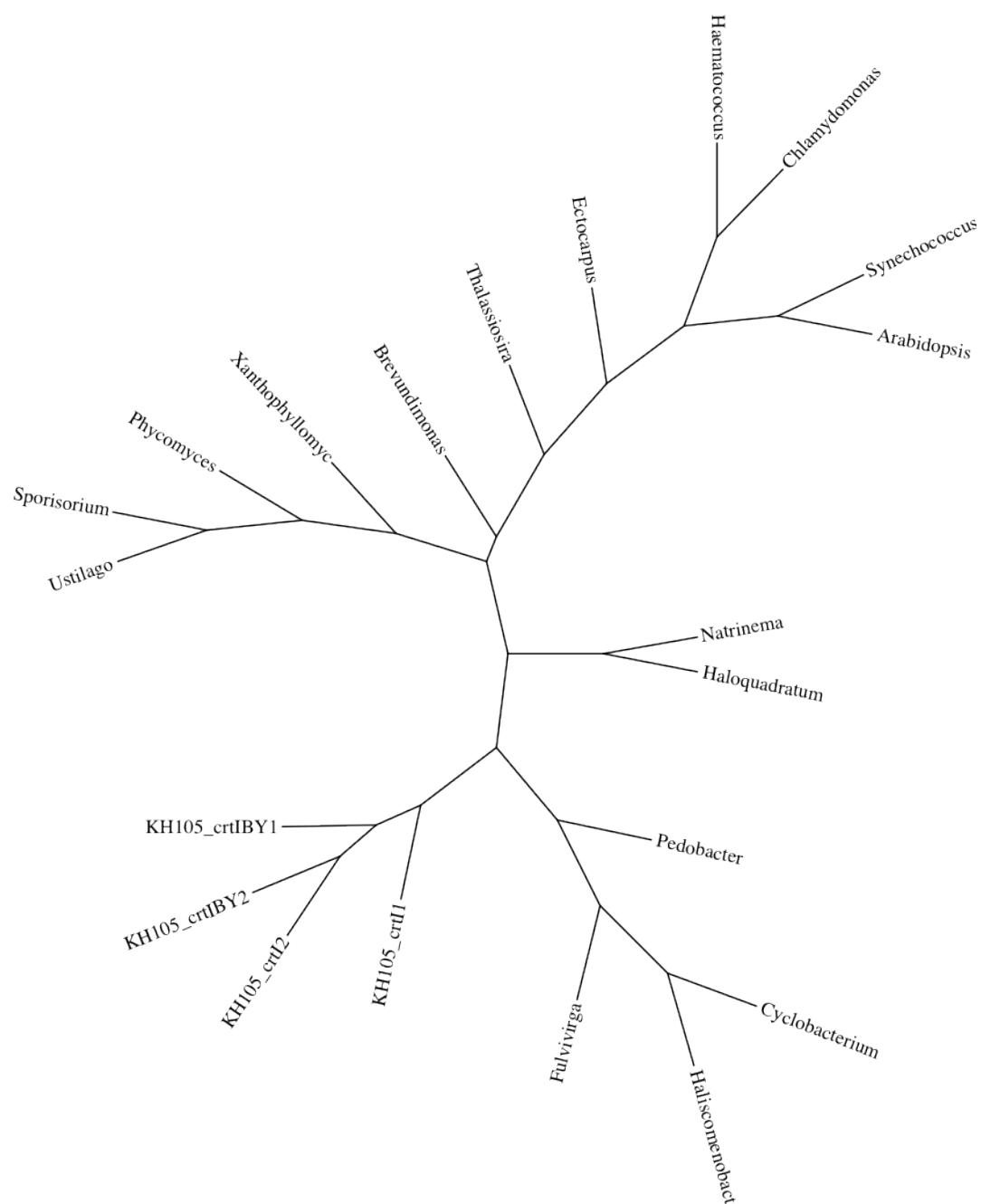

**Supplementary Figure S4:** Molecular phylogeny of KH105\_CrtI BY1, CrtI BY2, CrtI 1 and CrtI 2 by MP method.

Supplement: Supplementary file 1 [file genes-09-00200-s001.zip › Supplement/Fig. S4.pdf]

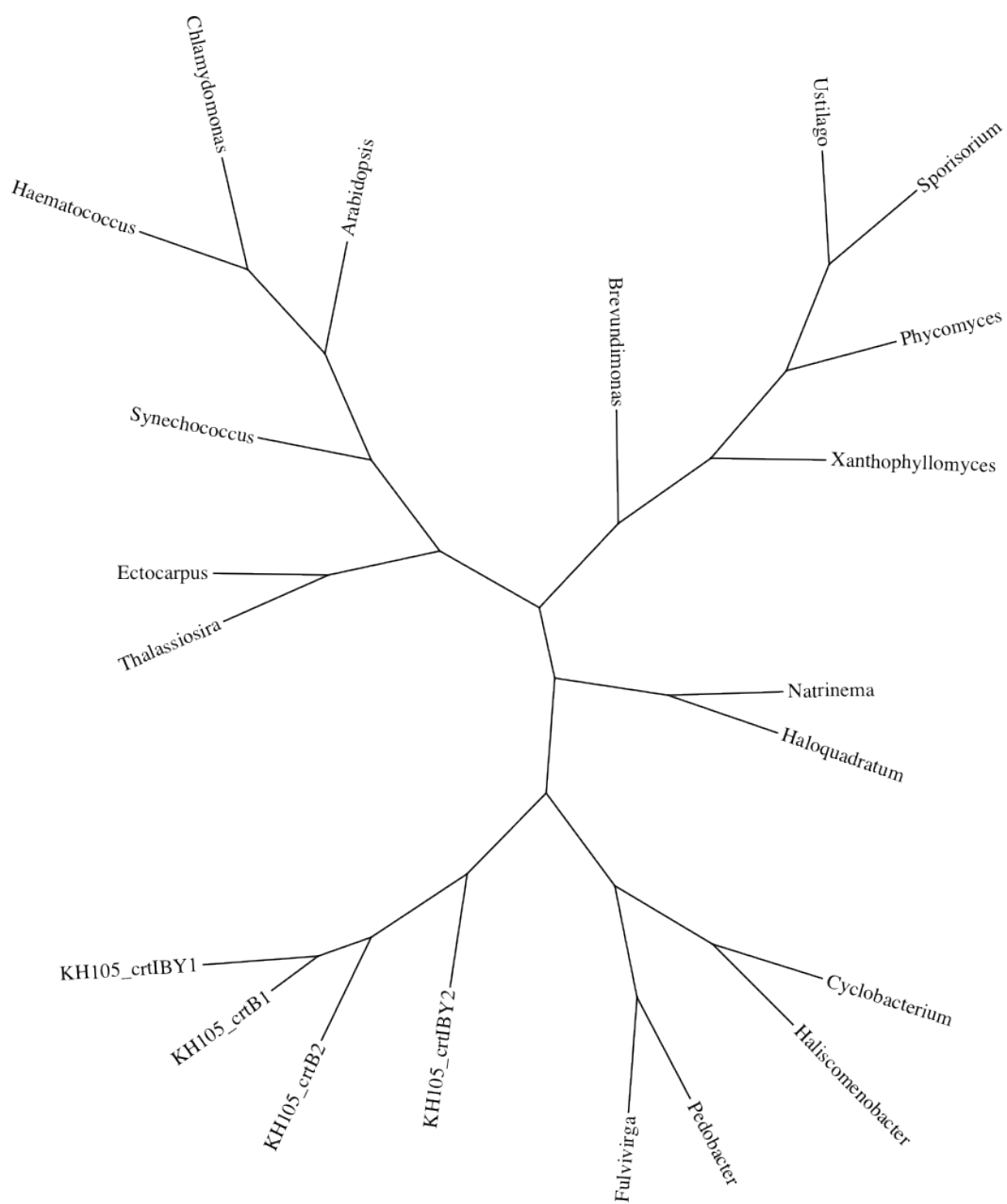

**Supplementary Figure S6:** Molecular phylogeny of KH105\_CrtI BY1, CrtI BY2, CrtB1 and CrtB2 by MP method.

Supplement: Supplementary file 1 [file genes-09-00200-s001.zip › Supplement/Fig. S6.pdf]

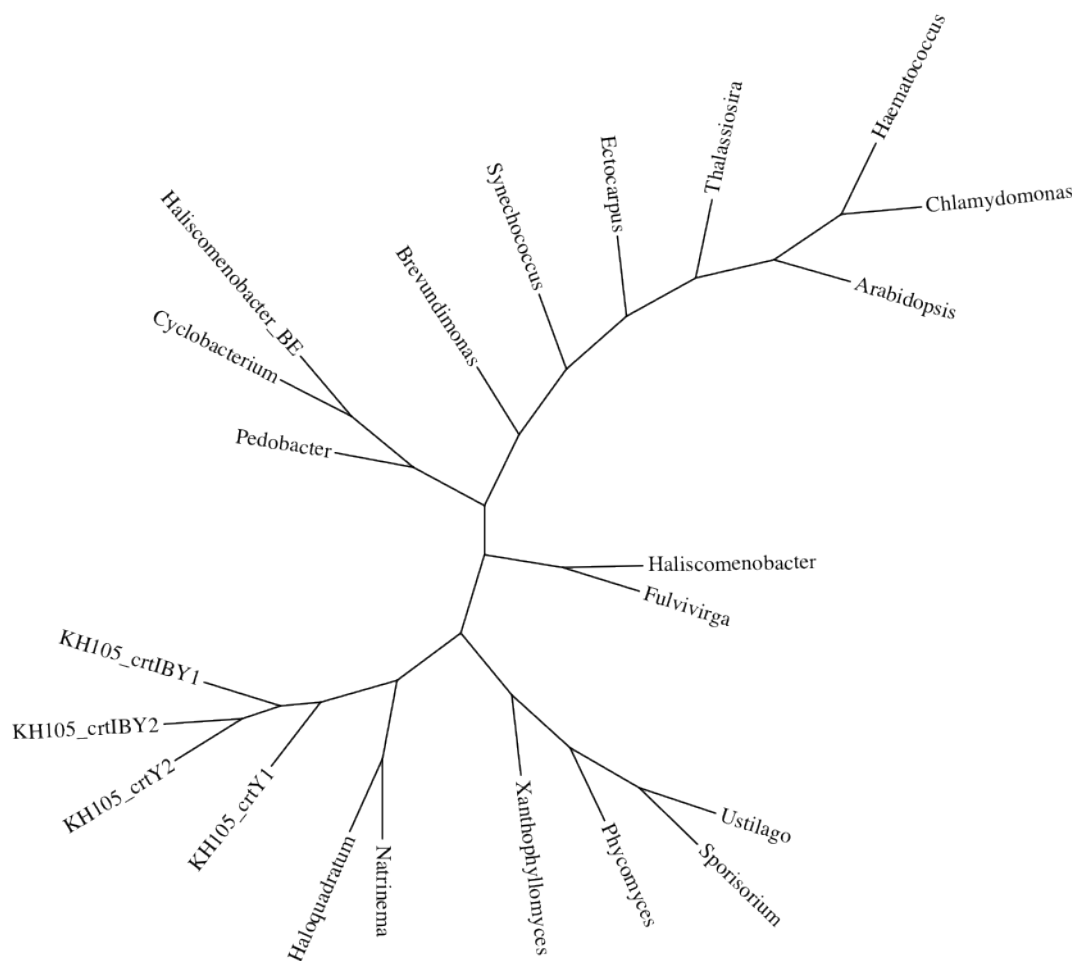

**Supplementary Figure S8:** Molecular phylogeny of KH105\_CrtIBY1, CrtIBY2, CrtB1 and CrtB2 by MP method.

Supplement: Supplementary file 1 [file genes-09-00200-s001.zip › Supplement/Fig. S8.pdf]
